# Efficacy of Injections with Disci/Rhus Toxicodendron Compositum for Chronic Low Back Pain – A Randomized Placebo-Controlled Trial

**DOI:** 10.1371/journal.pone.0026166

**Published:** 2011-11-08

**Authors:** Daniel Pach, Benno Brinkhaus, Stephanie Roll, Karl Wegscheider, Katja Icke, Stefan N. Willich, Claudia M. Witt

**Affiliations:** 1 Institute for Social Medicine, Epidemiology and Health Economics, Charité University Medical Center Berlin, Berlin, Germany; 2 Department of Medical Biometry and Epidemiology, University Medical Centre Hamburg-Eppendorf, Hamburg, Germany; 3 Center for Integrative Medicine, University of Maryland School of Medicine, Baltimore, Maryland, United States of America; Cochin Hospital (AP-HP), and the University Paris Descarte, France

## Abstract

**Background:**

The effectiveness of injection therapy for low-back pain is still debatable. We compared the efficacy of local injections of the homeopathic preparation Disci/Rhus toxicodendron compositum (verum) with placebo injections and with no treatment in patients with chronic low back pain.

**Methodology/Principal Findings:**

In a randomized controlled partly double blind multicenter trial patients with chronic low back pain from 9 German outpatient clinics were enrolled and randomly allocated in a 1∶1∶1 ratio to receive subcutaneous injections (verum or placebo) into painful sites on the lower back over 12 treatment sessions within eight weeks, or no treatment (rescue pain medication with paracetamol or NSAIDs). All trial personnel and participants were masked to treatment allocation. The primary outcome measure was the average pain intensity over the last seven days on a visual analogue scale (0–100 mm, 0 = no pain, 100 = worst imaginable pain) after eight weeks. Follow-up was 26 weeks. Primary analysis was by intention to treat. Between August 2007 and June 2008, 150 patients were randomly allocated to three groups (51 verum, 48 placebo and 51 no treatment). The mean baseline-adjusted low back pain intensity at week eight was: verum group 37.0 mm (97.5% CI 25.3;48.8), no treatment group 53.0 (41.8;64.2), and placebo group 41.8 (30.1;53.6). The verum was significantly superior to no treatment (P = 0.001), but not to placebo (P = 0.350). No significant side effects were reported.

**Conclusions/Significance:**

The homeopathic preparation was not superior to placebo. Compared to no treatment injections resulted in significant and clinical relevant chronic back pain relief.

**Trial Registration:**

ClinicalTrials.gov NCT00567736

## Introduction

In Western countries, chronic low back pain is a major health concern affecting quality of life and productivity. Low back pain has a high economic impact. More than 70% of the population in industrialised countries are affected by low back pain [1]. In the United Kingdom, low back pain accounts for 13% of absences due to illness. The annual incidence in adults is up to 45%, with those aged 35–55 years affected most often. Although 90% of episodes of acute low back pain settle within six weeks, up to 7% of patients develop chronic pain. For chronic low back pain, a wide range [2] of treatment options are available although their efficacy is not always clear. A multimodal approach is recommended including providing information and counseling, exercise, pain therapy, behavioral therapy, and physiotherapy [2], [3], [4]. However, long term effects are difficult to achieve [4]. Treatment with complementary and alternative medicine (CAM) therapies are widely used [5], [6], [7], [8], [9], [10].

Anthroposophic medicine is one of those CAM therapies. It was founded in the 1920s by Rudolf Steiner and Ita Wegman and aims to stimulate salutogenesis in patients by utilizing their self-healing capacities [11], [12]. It is practiced in around 67 countries around the world. Anthroposophic therapy for low back pain is provided by physicians (counseling, anthroposophic medication) and non-medical therapists (eurhythmy therapy, rhythmical massage therapy, embrocation, and art therapy). Anthroposophic drugs are of mineral, botanical or zoological origin, and are mostly used in homeopathic dilutions [11], [12].

The anthroposophic drug Disci/Rhus toxicodendron compositum (WALA Heilmittel GmbH) is used to treat acute low back pain. Some unpublished case reports of the manufacturer indicated that it might be effective for chronic low back pain. However, the effectiveness of injection therapy for low-back pain is still debatable [2], [13] and systematic data on the effectiveness or efficacy of Disci/Rhus toxicodendron compositum for chronic low back pain does not exist so far. The aim of the trial presented here was to determine the efficacy of Disci/Rhus toxicodendron compositum injections compared to placebo injections and no treatment in patients with chronic low back pain.

## Methods

### Design

A randomized controlled partly double-blind multicenter trial with a treatment duration of eight weeks and a follow-up after 26 weeks was performed to compare the injection of 10 ml Disci/Rhus toxicodendron compositum (verum) to 10 ml isotonic saline solution (placebo) and to a no treatment control. In the verum and in the placebo group both physicians and patients were blinded to group assignment. In addition, both participating statisticians were blinded for data analysis.

This study followed the standards of the Declaration of Helsinki, and the ICH-GCP guideline and was approved by the local ethics committees (Leading Ethics Committee in Berlin at the Landesamt für Gesundheit und Soziales, application No. 8031/07) and the Bundesinstitut für Arzneimittel und Medizinprodukte (application No. 61-3910-4032679). All patients gave written informed consent. The protocol for this trial and supporting CONSORT checklist are available as supporting information; see Checklist S1 and Protocol S1.

### Participants

Patients were recruited between August 2007 and June 2008 by nine study centers with various specializations (family medicine, internal medicine, orthopedics, rehabilitation, university outpatient clinics) in Germany. Participants in all three groups received the therapy free of charge (the no treatment group received the therapy after the study), but no allowance was paid. Participants were informed of the blinded study design and the randomized setting and the possibility of being assigned to the no treatment group. The central randomization sequence was generated with SAS 9.1 (SAS Institute, Cary, NC, USA) in a 1∶1∶1 ratio in blocks of ten stratified for centers. Randomization envelopes were prepared by two individuals who supervised each other and were not further involved in the study. They prepared opaque envelopes that were sequentially numbered and sealed, each containing a randomization number for each patient. Envelopes were opened by the study physician in consecutive order after gaining informed consent and baseline data. The number in the randomization envelope was identical with the patient code and the number on the medication box of the respective patient. Patients were eligible for the trial if they fulfilled the following inclusion criteria: age from 30 to 75 years, male or female, low back pain for at least 12 months (chronic low back pain), already received standard therapy, average back pain intensity of at least 40 mm on VAS (0–100 mm) in the last seven days at baseline, no other treatment except oral NSAIDs and muscle relaxants within four weeks prior to study entry, and informed consent. Women of childbearing potential were only included if they used effective contraceptive methods (Pearl Index <1).

Exclusion criteria included: previous or current treatment with Disci preparations, treatment other than NSAIDs or peripherally acting analgesics, routine use of analgesics for other diseases, protrusion or prolapsed intervertebral discs (one or more) with neurological symptoms, previous spinal surgery, suspected infectious spondylopathy, low back pain because of malignant or infectious disease, organic causes of back pain such as ankylosing spondylitis, Reiter syndrome and Behçet' syndrome, congenital deformities of the spine (without minor lordosis, kyphosis, scoliosis), suspected osteoporosis with compression fracture, suspected spinal stenosis, spondylolysis or spondylolisthesis, physiotherapy in the last four weeks prior or planned during the trial, the initiation of a new treatment for low back pain, complementary treatment in the last four weeks prior to or planned during the trial, inability to participate in the trial effectively, alcohol or substance abuse, participation in another clinical trial, severe chronic or acute disease which does not allow study participation, bleeding disorders or oral anticoagulation treatment, pregnancy and breast feeding, current application for a benefit, involvement in planning or coordination of the study, and hypersensitivity against drug components (Table 1).

**Table 1:** Postulated active ingredients of Disci/Rhus toxicodendron compositum.

Aconitum napellus e tubere ferm 33c Dil. D4	0.1 g
Argentum metallicum Dil. D18 aquos.	0.1 g
Arnica montana e planta tota ferm 33c Dil. D18	0.1 g
Disci intervertebrales bovis (cervicales, thoracici et lumbales) Gl Dil. D6	0.1 g
Formica rufa ex animale toto Gl Dil. D5	0.1 g
Gelsemium sempervirens e rhizoma ferm 35b Dil. D2	0.1 g
Granit Dil. D8	0.1 g
Leontopodium alpinum e planta tota ferm 36 Dil. D2	0.1 g
Mandragora officinarum e radice ferm 34d Dil. D4	0.1 g
Phyllostachys e nodo ferm 35c Dil. D4	0.1 g
Toxicodendron quercifolium e foliis ferm 33d Dil. D4	0.1 g

Other ingredients sodium chloride, sodium hydrogen carbonate, and water for injection.

### Intervention

Patients in the two treatment groups (verum and placebo) received 12 treatment sessions within eight weeks: twice per week for the first four weeks (with at least one day without therapy between sessions) and one treatment per week for the second four weeks (with at least three days without therapy between sessions). During each treatment session, 10 ml of solution was injected in 5 to 10 small dosages subcutaneously with a 0.4 mm needle into painful sites on the lower back. Disci/Rhus toxicodendron compositum is a composite medication based on the theory of anthroposophic medicine and is authorized in Germany. It consists of 11 different diluted agents (Table 1) and is traditionally used to treat disturbances of the spine, particularly acute pain associated with degenerative changes.

The placebo group received an injection with isotonic saline solution which contained sodium chloride, sodium hydrogen carbonate, and water and was not distinguishable from the verum solution. Patients in the no treatment group received no additional intervention during the study period. In all three groups, rescue pain medication with peripherally acting analgesics (also paracetamol) or NSAIDs, but not pain medication acting on the central nervous system, was permitted and their intake was documented in diaries.

### Outcome measurements

The primary outcome measure was the average low back pain intensity over the last seven days on a visual analogue scale [14] (VAS, 0–100 mm, 0 = no pain, 100 = worst imaginable pain) after eight weeks.

Secondary outcome measures included the VAS at 26 weeks, and the following outcomes at eight and 26 weeks: back function (Hannover Functional Ability Questionnaire, HFAQ; in German, Funktionsfragebogen Hannover Rücken), [15] quality of life (SF-36), [16] pain disability scale (PDI), [17] and pain perception scale (SES) [18], [19]. A patient diary (baseline to week 8) was used to calculate the number of days with medication between weeks five and eight. In addition, we evaluated the safety of the interventions and blinding (patient guess at 8 weeks).

To assess the patients' and doctors' expectation for improvement due to the treatment before randomization, patients and doctors had to document on categorical scales their expectation of the therapy: “recovery”, “distinct improvement”, “light improvement” and “no improvement”; as well as their assessment of the presumed therapy's effectiveness: “very effective”, “effective”, “small effect” and “no effect”.

### Statistical analysis

The primary analysis population was the intention to treat (ITT) population. Each randomized participant was included into the analysis regardless of the adherence to the assigned treatment or the provision of a full set of data. To detect a difference of 12.5 mm on the VAS between the verum group and the placebo group, with a pooled standard deviation of 18 mm (medium effect size according to Cohen d = 0.69) for the primary outcome measure with a power of 80% and an alpha-level of 2.5% (Bonferroni correction method to adjust for the testing of two primary analyses), a total of 123 participants were needed (41 per group). Taking about 20% potential drop outs into account, 150 participants were planned to be included into the study. For the primary analysis, a multilevel model with the two levels patient and study site was fitted to the data. The model was a linear mixed model with a random-effects parameter for study site, and additionally included the baseline VAS value as fixed covariate. Two primary hypotheses were tested comparing i) the verum group with the no treatment group and ii) the verum group with the placebo group. Both comparisons were made with the respective two-sided Wald test at an alpha level of 2.5% (Bonferroni correction) to set an overall significance level at 5%. From this model we estimated adjusted treatment effects and their confidence intervals (CI) at the 97.5%-level. Sensitivity analyses were performed by imputing missing values of the primary outcome using a maximum likelihood based imputation (regression method including baseline visual analogue scale, age and gender) and a “worst case scenario” method (where missing values in the verum group were imputed by the worst possible value (100) while missing values in the placebo and no treatment groups were imputed by the best possible value (0)). An additional per-protocol (PP) analysis was performed which included all randomized patients with complete primary outcome data (eight weeks), while excluding patients who had received less than 10 treatments during the first eight weeks or started a new therapy during the first eight weeks or received some kind of physiotherapy or CAM treatment during the first eight weeks. Patients were also excluded from the PP analysis if they required pain medication other than NSAIDs and peripherally acting analgesics or suffered from pseudospondylolisthesis. Further secondary analyses included an unadjusted analysis, the inclusion of other covariates into the primary model, and the analysis of all secondary outcomes with similar models (confidence intervals at the 95%-level for secondary outcomes).

## Results

### Participants

**Figure 1:**
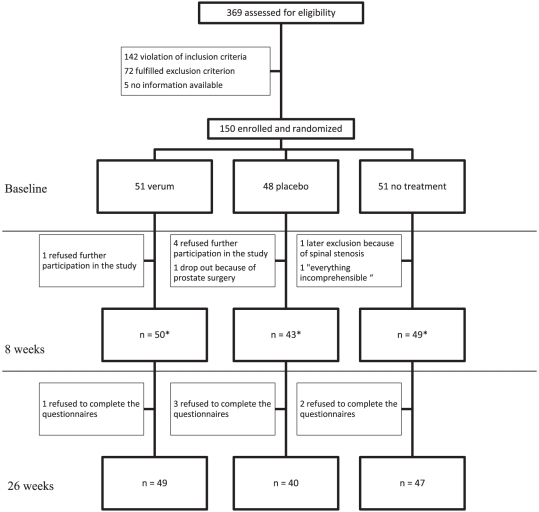
Trial flow chart. *primary outcome parameter available and used for primary analysis.

From 369 possible participants screened, 150 were enrolled and randomized into the three groups (verum group n = 51, placebo group n = 48, no treatment n = 51). The mean age was 57±11 (mean±sd) years, 64% were female and the mean duration of symptoms was 15±12 years. At baseline, the average pain intensity on the VAS was 60±14 mm (Table 2). 47 patients in the verum group and 42 patients in the placebo group received all 12 treatments (Figure 1). Eight patients were lost to follow-up at week eight, but were included in the ITT analysis. Follow-up data after 26 weeks was available for 136 patients (verum group n = 49, placebo group n = 40, no treatment group n = 47). The reasons for missing follow-up data are shown in Figure 1. For most baseline parameters, groups were comparable, with the exception of gender (P = 0.030), height (P = 0.014), and two scales of the SF-36, the physical component score (P = 0.010) and physical functioning subscale (P = 0.046). Expected treatment outcome was also comparable between the three groups.

**Table 2:** Baseline characteristics.

	Verum n = 51				No treatment n = 51				Placebo n = 48				
	mean±sd/n (%)			median	mean±sd/n (%)			median	mean±sd/n (%)			median	P value$
**Gender**													0.030
**Female**	40	(	78.4%	)	29	(	56.9%	)	27	(	56.3%	)	
**Male**	11	(	21.6%	)	22	(	43.1%	)	21	(	43.8%	)	
**Age (years)**	58.7	±	10.9	61.0	56.7	±	10.7	58.0	54.8	±	11.3	57.0	0.223
**Height (m)**	167.9	±	7.2	168.0	172.0	±	8.9	170.0	172.1	±	8.3	170.0	0.014
**Body weight (kg)**	75.0	±	13.9	74.0	77.1	±	12.9	76.0	78.2	±	15.6	76.0	0.524
**BMI (kg/m^2^)**	26.6	±	4.4	26.7	26.1	±	4.0	25.7	26.4	±	5.0	24.8	0.867
**Average pain intensity (VAS)**	58.9	±	14.3	54.0	59.0	±	14.1	55.0	62.5	±	13.9	61.5	0.360
**Medication intake**	22	(	43.1%	)	20	(	39.2%	)	15	(	31.3%	)	0.465
**Pain perception scale SES** #													
**Affective pain**	47.5	±	7.4	46.0	47.8	±	9.0	47.0	50.5	±	9.5	49.0	0.188
**Sensory pain**	47.5	±	8.4	46.0	49.4	±	9.0	49.0	49.6	±	9.7	48.0	0.447
**Pain disability index (PDI)** #	27.1	±	10.7	26.0	27.7	±	11.8	26.5	29.0	±	13.8	28.0	0.741
**Back function (HFAQ)** *	61.9	±	18.0	66.7	65.5	±	17.4	66.7	61.3	±	21.8	62.5	0.491
**SF-36 quality of life** *													
**Physical Component Score**	36.2	±	6.3	36.5	35.1	±	7.7	35.2	31.6	±	8.9	30.5	0.010
**Mental Component Score**	48.8	±	12.3	54.1	49.2	±	11.0	53.3	50.5	±	11.5	53.6	0.756
**Physical functioning**	60.5	±	15.4	60.0	59.0	±	22.5	57.5	50.8	±	23.0	50.0	0.046
**Role physical**	47.6	±	38.5	50.0	35.0	±	35.4	25.0	33.9	±	35.9	25.0	0.119
**Bodily pain**	37.0	±	14.4	41.0	37.0	±	13.9	41.0	31.7	±	14.8	31.0	0.111
**General health perception**	53.7	±	17.2	53.5	56.6	±	18.0	55.0	50.8	±	19.6	47.0	0.304
**Vitality**	48.6	±	18.6	45.0	45.6	±	19.2	45.0	45.3	±	19.6	45.0	0.630
**Social functioning**	74.5	±	24.2	75.0	74.8	±	21.9	75.0	71.1	±	24.4	75.0	0.692
**Role emotional**	68.0	±	41.6	100.0	63.3	±	44.7	100.0	71.5	±	41.8	100.0	0.634
**Mental health**	68.0	±	21.1	76.0	70.0	±	17.5	76.0	67.4	±	20.0	72.0	0.789
**Effectiveness of the therapy with the verum (physician)**													0.482μ
**Very effective**	4	(	7.8%	)	6	(	11.8%	)	3	(	6.3%	)	
**Effective**	41	(	80.4%	)	41	(	80.4%	)	39	(	81.3%	)	
**Small effect**	6	(	11.8%	)	4	(	7.8%	)	6	(	12.5%	)	
**No effect**	0	(	0.0%	)	0	(	0.0%	)	0	(	0.0%	)	
**Effectiveness of the therapy with the verum (patient)**													0.706μ
**Very effective**	7	(	14.0%	)	10	(	20.4%	)	8	(	17.8%	)	
**Effective**	38	(	76.0%	)	35	(	71.4%	)	32	(	71.1%	)	
**Small effect**	4	(	8.0%	)	4	(	8.2%	)	5	(	11.1%	)	
**No effect**	1	(	2.0%	)	0	(	0.0%	)	0	(	0.0%	)	
**Expectation of the therapy with the verum (physician)**													0.389μ
**Recovery**	3	(	5.9%	)	3	(	5.9%	)	2	(	4.2%	)	
**Distinct improvement**	21	(	41.2%	)	23	(	45.1%	)	16	(	33.3%	)	
**Light improvement**	27	(	52.9%	)	24	(	47.1%	)	29	(	60.4%	)	
**No improvement**	0	(	0.0%	)	1	(	2.0%	)	1	(	2.1%	)	
**Expectation of the therapy with the verum (patient)**													0.358μ
**Recovery**	7	(	13.7%	)	5	(	9.8%	)	2	(	4.2%	)	
**Distinct improvement**	38	(	74.5%	)	44	(	86.3%	)	51	(	85.4%	)	
**Light improvement**	5	(	9.8%	)	2	(	3.9%	)	5	(	10.4%	)	
**No improvement**	1	(	2.0%	)	0	(	0.0%	)	0	(	0.0%	)	

$ one-way ANOVA,

# lower values are better,

*higher values are better.

μ Kruskal-Wallis test.

### Outcome measures

**Figure 2:**
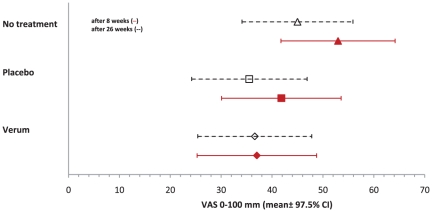
Mean (with 95% confidence interval) pain intensity over the last 7 days (VAS) at week 8 (primary outcome) and at week 26. Differences were statistically significant for the comparison of verum and no treatment group at 8 weeks (P<0.001), but not for the comparison with the placebo group (P = 0.350) and at 26 weeks for both group comparisons (P = 0.085 and P = 0.837, respectively).

Figure 2 shows the result for the primary outcome measure, the adjusted mean VAS for average low back pain intensity in the last seven days at week eight. Average pain was 37.0 [97.5% CI 25.3;48.8] in the verum group, 53.0 [41.8;64.2] in the no treatment group, and 41.8 [30.1;53.6] in the placebo group. The VAS was statistically significant lower in the verum group than in the no treatment group (P = 0.001, also Table 3), but no significant differences could be shown between the verum and the placebo group (P = 0.350). Unadjusted analysis, per-protocol analysis, and analyses with the imputation of missing values yielded similar results (Table 4). Moreover, the inclusion of other covariates in the model such as gender, which was significant different at baseline, did not change the results. According to Cohen's d the effect size for the comparison between verum and no treatment group was moderate (0.68).[20]

**Table 3:** Outcome measures§.

	Verum n = 50				No treatment n = 49				Placebo n = 43				Verum vs no treatment	Verum vs placebo
	mean	95%CI			mean	95%CI			mean	95%CI			P value	P value
**Average pain intensity (VAS)**														
**8 weeks (97.5% CI)**	37.0	25.3	;	48.8	53.0	41.8	;	64.2	41.8	30.1	;	53.6	0.001	0.350
**26 weeks**	36.6	25.4	;	47.8	45.0	34.1	;	55.9	35.5	24.2	;	46.9	0.085	0.837
**Days with rescue medication**														
**Week 1–4**	3.9	1.1	;	6.8	8.8	6.0	;	11.6	2.8	−0.1	;	5.7	<0.001	0.396
**Week 5–8**	3.7	1.2	;	6.3	8.2	5.7	;	10.7	3.3	0.8	;	5.9	0.001	0.785
**Week 1–8**	7.7	2.5	;	12.9	17.1	12.0	;	22.2	6.0	0.7	;	11.4	<0.001	0.532
**Pain perception scale (SES)** #														
**Affective pain**														
**8 weeks**	44.0	41.7	;	46.3	44.9	42.5	;	47.3	43.5	41.0	;	46.1	0.590	0.795
**26 weeks**	42.9	40.0	;	45.7	42.1	39.3	;	45.0	41.4	38.3	;	44.4	0.686	0.420
**Sensory pain**														
**8 weeks**	45.3	43.3	;	47.3	45.0	43.0	;	47.0	46.1	44.0	;	48.2	0.811	0.594
**26 weeks**	45.5	42.8	;	48.1	44.8	42.2	;	47.4	43.7	41.0	;	46.3	0.680	0.277
**Pain disability index (PDI)** #														
**8 weeks**	22.7	19.3	;	26.2	25.9	22.5	;	29.3	21.4	17.7	;	25.1	0.200	0.598
**26 weeks**	18.1	14.0	;	22.3	22.7	18.7	;	26.7	21.4	17.2	;	25.6	0.046	0.173
Back function (HFAQ)*														
**8 weeks**	68.3	64.0	;	72.6	64.8	60.5	;	69.1	68.4	63.8	;	73.0	0.261	0.969
**26 weeks**	69.0	62.8	;	75.2	64.8	58.8	;	70.9	67.4	61.0	;	73.8	0.226	0.660
SF-36 quality of life*														
**Physical component score**														
**8 weeks**	37.1	34.9	;	39.2	35.4	33.3	;	37.5	39.8	37.5	;	42.1	0.278	0.089
**26 weeks**	38.2	35.0	;	41.5	36.5	33.3	;	39.7	40.9	37.5	;	44.2	0.326	0.163
**Mental component score**														
**8 weeks**	48.5	46.0	;	50.9	50.9	48.4	;	53.3	47.5	44.9	;	50.1	0.174	0.609
**26 weeks**	51.2	48.9	;	53.5	51.5	49.1	;	53.9	48.9	46.4	;	51.4	0.861	0.185
**Physical functioning**														
**8 weeks**	59.6	55.2	;	64.1	59.8	55.3	;	64.3	64.0	59.2	;	68.9	0.955	0.196
**26 weeks**	63.4	56.7	;	70.0	60.1	53.6	;	66.6	66.3	59.5	;	73.2	0.370	0.439
**Role physical**														
**8 weeks**	47.8	38.3	;	57.3	47.1	37.7	;	56.6	57.0	46.8	;	67.2	0.919	0.198
**26 weeks**	54.7	42.0	;	67.3	49.7	37.3	;	62.1	60.5	47.4	;	73.7	0.508	0.458
**Bodily pain**														
**8 weeks**	48.0	42.6	;	53.5	40.0	34.5	;	45.5	46.8	40.9	;	52.7	0.041	0.767
**26 weeks**	53.3	45.2	;	61.4	46.1	38.1	;	54.0	50.2	41.9	;	58.5	0.085	0.483
**General health perception**														
**8 weeks**	53.7	49.7	;	57.7	52.9	48.9	;	56.9	54.2	49.9	;	58.5	0.773	0.878
**26 weeks**	54.8	50.2	;	59.4	51.9	47.2	;	56.5	57.1	52.1	;	62.1	0.321	0.465
**Vitality**														
**8 weeks**	45.5	41.0	;	50.0	44.5	40.0	;	49.0	51.1	46.3	;	56.0	0.759	0.096
**26 weeks**	50.1	45.0	;	55.3	49.2	44.2	;	54.3	51.7	46.3	;	57.0	0.764	0.614
**Social functioning**														
**8 weeks**	73.9	68.5	;	79.3	76.7	71.3	;	82.2	75.4	69.6	;	81.3	0.472	0.712
**26 weeks**	81.5	76.5	;	86.5	78.2	73.0	;	83.3	78.7	73.2	;	84.3	0.363	0.470
**Role emotional**														
**8 weeks**	75.5	65.9	;	85.1	74.4	64.5	;	84.3	62.5	52.1	;	72.9	0.874	0.072
**26 weeks**	80.8	71.7	;	89.9	80.7	71.2	;	90.1	71.6	61.4	;	81.7	0.982	0.182
**Mental health**														
**8 weeks**	64.9	60.7	;	69.1	70.9	66.7	;	75.2	68.2	63.7	;	72.8	0.047	0.283
**26 weeks**	70.2	65.8	;	74.6	70.1	65.6	;	74.6	67.9	63.0	;	72.8	0.970	0.487

§ data were adjusted using a linear mixed model with a random-effects parameter for study site, and the respective baseline value as fixed covariate.

# lower values are better, *higher values are better.

**Table 4:** Sensitivity and additional analyses of the visual analogue scale (VAS), unadjusted data, per-protocol analysis, analysis with imputation of missing values and models with additional covariates.

	Verum		No treatment		Placebo		Verum vs no treatment	Verum vs placebo
	mean VAS	CI	mean VAS	CI	mean VAS	CI	P value	P value
**Unadjusted analysis**								
**8 weeks (97.5% CI)**	36.6	27.8;45.4	52.6	46.2;59.1	43.4	33.3;53.4	0.001	0.244
**26 weeks (95% CI)**	37.5	29.7;45.4	46.1	39.2;53.1	38.5	30.7;46.3	0.104	0.858
**Per-protocol analysis**								
**8 weeks (97.5% CI)**	41.5	29.6;53.3	52.9	41.8;64.1	37.8	25.9;49.8	0.035	0.518
**26 weeks (95% CI)**	38.5	27.2;49.7	45.2	34.3;56.0	36.5	25.3;47.7	0.232	0.732
**Imputation of missing values VAS at 8 weeks (97.5% CI)**								
**Maximum likelihood method** *	37.2	27.7;46.8	52.7	43.3;62.1;	42.3	32.1;52.4	0.002	0.329
**Worst case method** #	38.4	30.1;46.7	51.0	42.7;59.3	37.8	29.2;46.4	0.016	0.921
**Primary model with other additional covariates VAS at 8 weeks (97.5% CI)**								
**Gender**	37.1	25.2;48.9	53.0	41.8;64.2	41.8	30.1;53.6	0.002	0.367
**Expectation of the therapy with the verum (patient)**	33.5	21.0;45.9	48.7	35.9;61.5	38.2	25.5;50.9	0.002	0.360
**Expectation of the therapy with the verum (physician)**	36.9	24.9;49.0	52.8	41.2;64.5	41.8	29.9;53.7	0.002	0.346

*regression analysis including the baseline VAS, age and gender.

# missing values in the verum group were imputed by the worst possible value (100) while missing values in the placebo and no treatment groups were imputed by the best possible value (0).

After 26 weeks the average pain severity did not differ significantly between the three groups and was lower than after 8 weeks (Table 3). A trend towards a difference between the verum 36.6 [95% CI 25.4;47.8] and the no treatment group 45.0 [34.1;55.9] after 26 weeks was revealed (P = 0.085).

For most of the secondary outcomes after 8 weeks and 26 weeks, no significant group differences were observed with the exception of some differences between the verum and the no treatment group regarding rescue medication, pain disability index after 26 weeks, and quality of life subscales. For example, in the no treatment group rescue medication was used on more days during the weeks five to eight (8.2 [5.7;10.7] days) than in the verum group (3.7 [1.2;6.3] days, P = 0.001). However, results in the verum and the placebo group for rescue medication and all other secondary outcome parameters were not significantly different (Table 3).

Of the 99 patients in both intervention groups, 71 patients reported at least one adverse event (verum group n = 37, placebo group n = 34). Reported adverse events included a hematoma at the injection site (verum group n = 8 (15.7%) vs. placebo group n = 5 (10.4%), P = 0.546), common cold (9 (17.6%) vs. 5 (10.4%), P = 0.379) and pain (17 (33.3%) vs. 17 (35.4%), P = 0.814). We did not observe any significant or relevant differences between both groups.

After eight weeks of treatment, both patients and physicians were asked to guess what treatment intervention had been administered to each patient (Table 5). Treatment with Disci/Rhus toxicodendron. compositum could not be identified more often than expected by chance. The allocation of each guess did not differ significantly between the treatment groups (physicians P = 0.292, patients P = 0.255).

**Table 5:** Guesses of group allocation as a surrogate for blinding.

	Group assignment		P value*
Physicians' guesses	Verum	Placebo	
**Verum**	28 (59.6%)	20 (47.6%)	0.292
**Placebo**	19 (40.4%)	22 (52.4%)	

Patients' guesses	Verum	Placebo	P value*
**Verum**	14 (28.0%)	9 (20.9%)	0.255
**Placebo**	20 (40.0%)	13 (30.2%)	
**“I don't know”**	16 (32.0%)	21 (48.8%)	

*Chi-square test.

Neither treatment expectation of the patients nor the physicians had a significant influence on the patients' outcome.

## Discussion

In our study, we found that the homeopathic preparation Disci/Rhus toxicodendron compositum was not superior to placebo. Compared to no treatment the injection treatment resulted in significant and clinically relevant short-term chronic back pain relief and reduction of rescue medication. In addition, it was safe.

The main strengths of this trial are the double-blind randomized placebo controlled design with a multicenter approach, the inclusion of a no treatment group, the relatively large sample size for a trial on CAM, and the good compliance and follow-up rates. Concealed treatment allocation and sustained blinding in the treatment groups could be ensured for both doctors and patients. Patients were recruited and treated at very different study sites with different clinical settings such as university outpatient clinics, primary care practices, and orthopedic practices to ensure better external validity of the results. With the three armed design we were able to evaluate the impact of the whole intervention (verum group vs. no treatment group) as well as the specific effect of the drug alone (verum group vs. placebo group).

The primary outcome measure (VAS) is a validated and sensitive tool which is widely used to measure pain. The VAS was measured after eight weeks and displays the effect of the intervention at the end of a treatment phase. In addition we evaluated long term effects after 26 weeks. We included a number of secondary outcome measures such as medication intake, back function and quality of life. Those together with the sensitivity and per protocol analyses help to confirm the results.

The placebo control we used was an isotonic saline solution which could not be distinguished from the verum. Like in the verum group, at every treatment session the isotonic solution was injected subcutaneously into the lower back. One may argue that physiological effects caused by the insertion of the needle and the injection of a solution cannot be ruled out. For example, according to acupuncture research those effects could have been mediated by diffuse noxious inhibitory control [21], [22]. Thus, the injections themselves, even without an active ingredient, can affect pain perception. Consequently, according to Kaptchuk the placebo control we used can be described as very powerful [23]. A recent study on acupuncture shows that pricking the skin without penetration can reduce pain in low back pain patients [24]. Our study had the power to detect a clinically relevant difference on VAS between the verum and no treatment (according Ostelo [25] around 15 mm). However, between the verum and the placebo group no significant difference was shown on the VAS which might only be statistically significant in a much larger sample.

Another limitation of this trial might be the therapy duration that we chose. We evaluated the effect of 12 therapy sessions within eight weeks. This duration of the therapy might be too short to cause substantial effects in patients with long term chronic low back pain of 15 years. Moreover, today a multimodal approach for patients with chronic low back pain without injection therapy is recommended [2], [3], [4].

As this was the first prospective study on Disci/Rhus toxicodendron compositum in patients with chronic low back pain we lack the possibility to compare our results with results from other studies. The effectiveness of injection therapy in treating low back pain independent of the injected solution was shown by a study with 110 patients who received either glucose-lignocaine or saline injections [26]. And according to a Cochrane review on injection therapy for subacute and chronic low back pain there is no strong evidence for or against the use of any type of injection therapy [13]. Another Cochrane review, on acupuncture and dry-needling for low back pain, concludes that acupuncture and dry-needling may be useful adjuncts to other therapies for chronic low-back pain [27]. A systematic review [28] from 2008 indicates that the injection of sterile water can be used as a treatment option for low back pain during labor. Sterile water seems to be more effective than isotonic solutions [29] which might be explained by an osmotic irritation as well as mechanical stimulation in the injection area because of the increased local pressure in the tissue, a kind of sensory stimulation [28]. Local anti-nociceptive effects mediated by adenosine A1 receptors as recently shown for acupuncture by Goldman et al. in Nature Neuroscience are also possible [30]. This suggests that injections can have strong specific effects. Moreover, local subcutaneous injections of safe substances such as water and saline solution might have its role in the treatment of low back pain and further research would be helpful. We conclude that no superiority of Disci/Rhus toxicodendron compositum over placebo injections could be shown for patients with chronic low back pain. However, injection therapy was safe and a short term reduction of pain and rescue medication was achieved by subcutaneous injections of both verum and placebo when compared to no treatment. The role of local subcutaneous injections of safe substances such as water and saline solution for low back pain management should be further investigated.

## Supporting Information

Protocol S1 Trial protocol.
(DOC)

Checklist S1 CONSORT checklist.
(DOC)
