# Supplementary material for: Efficacy of Injections with Disci/Rhus Toxicodendron Compositum for Chronic Low Back Pain – A Randomized Placebo-Controlled Trial
Source: PLoS One. 2011 Nov 8;6(11):e26166. doi: 10.1371/journal.pone.0026166 (PMC3210748; doi:10.1371/journal.pone.0026166)
Supplement: Protocol S1 — Trial protocol. (DOC) [file pone.0026166.s001.doc]

| EudraCT-Nr.: | 2006-006390-24 | | |
| --- | --- | --- | --- |
| Studien-Code: | DISCI-Studie | | |
| Studiencode (Sponsor): | disrhu-1 | | |
| Datum des  Prüfplans: | 19.03.2007 | | |
| Version-Nr.: | 02 | | |
|  | |  |  |
|  | | | |
|  | | | |
| **Prospektive, randomisierte, kontrollierte, multizentrische, teils doppelblinde Studie zum Vergleich von Disci/Rhus toxicodendron comp.®, Placebo und Warteliste bei Patienten mit chronischen LWS-Schmerzen**  DISCI-Studie  PRÜFPLAN | | | |
|  | | | |
|  | | | |


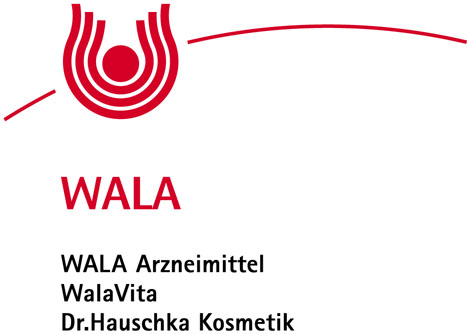

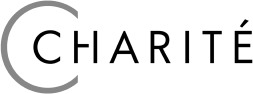


Charité – Universitätsmedizin Berlin

Institut für Sozialmedizin, Epidemiologie

und Gesundheitsökonomie

_________________________________________________________________________________

**Änderungen gegenüber Prüfplan Version 01_19.12.2006**

| **Inhaltsverzeichnis** | **Änderung gegenüber Version 01_19.12.2006** |
| --- | --- |
| Synopsis Punkt 6: Ziel- Patientenpopulation:  Einschlusskriterien Punkt 4 | Schmerzen der LWS seit mindestens 12 Monaten, die therapeutischen Standardtherapiemaßnahmen müssen ausgeschöpft sein |
| Synopsis Punkt 6: Ziel- Patientenpopulation:  Einschlusskriterien Punkt 7 | Es gelten kontrazeptive Maßnahmen als effektiv, wenn die Versagerate <1%/Jahr beträgt, z.B. orale hormonelle Verhütung mit Kombinationspräparaten, Implantate, Intrauterinpessar, Diaphragmapessar, Portioklappe oder sexuelle Abstinenz. |
| Synopsis Punkt 6: Ziel- Patientenpopulation:  Ausschlusskriterien Punkt 21 | „schwerwiegend“ wird gestrichen |
| Synopsis Punkt 8: Testdauer | Satz: “Anschließend wird den Patienten der Wartelistengruppe eine Disci/Rhus toxicodendron comp.®-Behandlung außerhalb der Studienevaluation angeboten.“ wird gestrichen |
| Punkt 4.2: Einschlusskriterien Punkt 4 | Schmerzen der LWS seit mindestens 12 Monaten, die therapeutischen Standardtherapiemaßnahmen müssen ausgeschöpft sein |
| Punkt 4.2: Einschlusskriterien Punkt 7 | Es gelten kontrazeptive Maßnahmen als effektiv, wenn die Versagerate <1%/Jahr beträgt, z.B. orale hormonelle Verhütung mit Kombinationspräparaten, Implantate, Intrauterinpessar, Diaphragmapessar, Portioklappe oder sexuelle Abstinenz. |
| Punkt 4.3: Ausschlusskriterien Punkt 21 | „schwerwiegend“ wird gestrichen |

Weitergabe, Vervielfältigung oder Verwendung in Publikationen nur mit
schriftlicher Genehmigung des Sponsors und des Institutes

***Inhaltsverzeichnis***

[SYNOPSIS 6](#__RefHeading___Toc154297772)

[Flow Chart für den Arzt 13](#__RefHeading___Toc154297773)

[Liste der Abkürzungen und Definitionen 14](#__RefHeading___Toc154297774)

[Liste Der Wichtigsten Formulare 15](#__RefHeading___Toc154297775)

[Nomenklatur im Prüfplan 16](#__RefHeading___Toc154297776)

[PRÜFÄRZTE UND VERANTWORTLICHE 17](#__RefHeading___Toc154297777)

[1. Einleitung 22](#__RefHeading___Toc154297778)

[1.1 Allgemeiner Hintergrund 22](#__RefHeading___Toc154297779)

[1.2 Hintergrund der klinischen Prüfung und Fragestellung 22](#__RefHeading___Toc154297780)

[2. Studienziele 23](#__RefHeading___Toc154297781)

[2.1 Primäres Ziel und primäre Zielgröße 23](#__RefHeading___Toc154297782)

[2.2 Sekundäre Ziele und sekundäre Zielgrößen 23](#__RefHeading___Toc154297783)

[2.3 Nutzen – Risiko-Bewertung der Studie 24](#__RefHeading___Toc154297784)

[3. Studiendesign 25](#__RefHeading___Toc154297785)

[3.1 Hintergründe zum Studiendesign, Dosis und Kontrollgruppe 25](#__RefHeading___Toc154297786)

[3.2 Randomisierung 26](#__RefHeading___Toc154297787)

[4. Auswahl der Studienpopulation 26](#__RefHeading___Toc154297788)

[4.1 Rekrutierung 26](#__RefHeading___Toc154297789)

[4.2 Einschlusskriterien 27](#__RefHeading___Toc154297790)

[4.3 Ausschlusskriterien 27](#__RefHeading___Toc154297791)

[4.4 Erläuterung der Auswahlkriterien der Studienteilnehmer 28](#__RefHeading___Toc154297792)

[4.5 Erläuterung zur Geschlechterverteilung der Studienteilnehmer 29](#__RefHeading___Toc154297793)

[4.6 Abbruch der klinischen Studie beim Studienteilnehmer 29](#__RefHeading___Toc154297794)

[4.6.1 Abbruchkriterien 29](#__RefHeading___Toc154297795)

[4.6.2 Vorgehen bei vorzeitigem Therapie-/Studienabbruch 29](#__RefHeading___Toc154297796)

[4.6.3 Verfahren nach dem individuellen Studienende 30](#__RefHeading___Toc154297797)

[5. Prüfpräparate 30](#__RefHeading___Toc154297798)

[5.1 Prüfpräparate 30](#__RefHeading___Toc154297799)

[5.1.1 Beschreibung und Zusammensetzung der Prüfpräparate 30](#__RefHeading___Toc154297800)

[5.1.1.1 Disci/Rhus toxicodendron comp.® (Verum und Warteliste) 30](#__RefHeading___Toc154297801)

[5.1.1.2 Isotone Lösung (Placebo) 31](#__RefHeading___Toc154297802)

[5.1.2 Art der Anwendung 31](#__RefHeading___Toc154297803)

[5.1.3 Herstellung 31](#__RefHeading___Toc154297804)

[5.1.4 Kennzeichnung der Prüfpräparate 31](#__RefHeading___Toc154297805)

[5.1.5 Verpackung und Bezug der Prüfpräparate 32](#__RefHeading___Toc154297806)

[5.1.6 Lagerung 32](#__RefHeading___Toc154297807)

[5.1.7 Umgang mit den Prüfpräparaten 33](#__RefHeading___Toc154297808)

[5.1.8 Bilanzierung (Drug Accountability) 33](#__RefHeading___Toc154297809)

[5.1.9 Randomisierung 33](#__RefHeading___Toc154297810)

[5.1.10 Verblindung 33](#__RefHeading___Toc154297811)

[5.1.11 Entblindung 34](#__RefHeading___Toc154297812)

[5.1.12 Nebenwirkungen, Wechselwirkungen und Gegenanzeigen 34](#__RefHeading___Toc154297813)

[5.2 Begleitbehandlung (vor-, während-, nach der klinischen Prüfung) 34](#__RefHeading___Toc154297814)

[5.3 Compliance 35](#__RefHeading___Toc154297815)

[6. Dokumentationsinstrumente 35](#__RefHeading___Toc154297816)

[6.1 Pre-Study Screening Log 36](#__RefHeading___Toc154297817)

[6.2 Einwilligungserklärung 36](#__RefHeading___Toc154297818)

[6.3 Patientenidentifikation 36](#__RefHeading___Toc154297819)

[6.4 Faxmeldung „Patienteneinschluss“ 36](#__RefHeading___Toc154297820)

[6.5 Schmerzskala (VAS) 36](#__RefHeading___Toc154297821)

[6.6 Einschlussuntersuchung 37](#__RefHeading___Toc154297822)

[6.7 Therapiedokumentationen 37](#__RefHeading___Toc154297823)

[6.8 Befragung nach der 3. Behandlung 37](#__RefHeading___Toc154297824)

[6.9 Patiententagebuch 37](#__RefHeading___Toc154297825)

[6.10 Patientenfragebögen 1-3 37](#__RefHeading___Toc154297826)

[6.11 Entblindung 38](#__RefHeading___Toc154297827)

[6.12 Therapie- und Studienabbruch 38](#__RefHeading___Toc154297828)

[6.13 Bericht über UE 38](#__RefHeading___Toc154297829)

[6.14 Bericht über SUE 38](#__RefHeading___Toc154297830)

[7. Studienablauf 39](#__RefHeading___Toc154297831)

[7.1 Zeitplan 39](#__RefHeading___Toc154297832)

[7.2 Studieneinschluss und Erhebung der Baselinedaten 39](#__RefHeading___Toc154297833)

[7.2.1 Vergabe der Pre-Study Screeningnummer 39](#__RefHeading___Toc154297834)

[7.2.2 Aufklärung und Einholung der Einwilligung 39](#__RefHeading___Toc154297835)

[7.2.3 Erhebung der Anamnese und Einschlussuntersuchungen 40](#__RefHeading___Toc154297836)

[7.2.4 Überprüfung der Ein- und Ausschlusskriterien 40](#__RefHeading___Toc154297837)

[7.2.5 Patientenfragebogen 1 40](#__RefHeading___Toc154297838)

[7.2.6 Randomisierung und Vergabe des Patientencodes 40](#__RefHeading___Toc154297839)

[7.2.7 Patiententagebuch 1 41](#__RefHeading___Toc154297840)

[7.3 Behandlungsphase (Woche 1-8) in Verum- und Placebogruppe 41](#__RefHeading___Toc154297841)

[7.3.1 Therapiedokumentation 41](#__RefHeading___Toc154297842)

[7.3.2 Befragung nach der 3. Behandlung 41](#__RefHeading___Toc154297843)

[7.3.3 Patiententagebücher 2 und 3 41](#__RefHeading___Toc154297844)

[7.3.4 Patientenfragebogen 2 42](#__RefHeading___Toc154297845)

[7.4 Wartephase (Woche 1-8) in Wartelistengruppe 42](#__RefHeading___Toc154297846)

[7.4.1 Patiententagebücher 2 und 3 42](#__RefHeading___Toc154297847)

[7.4.2 Patientenfragebogen 2 42](#__RefHeading___Toc154297848)

[7.5 Follow up (nach 26 Wochen) 42](#__RefHeading___Toc154297849)

[7.5.1 Patientenfragebogen 3 42](#__RefHeading___Toc154297850)

[7.5.2 Behandlungsphase für die Wartelistengruppe (Woche 26 - 34) 42](#__RefHeading___Toc154297851)

[8. Sicherheitsparameter und Variablen 43](#__RefHeading___Toc154297852)

[8.1 Definition unerwünschtes Ereignis UE (gemäß GCP-V/ ICH-GCP) 43](#__RefHeading___Toc154297853)

[8.2 Definition schwerwiegendes unerwünschtes Ereignis SUE (gemäß GCP-V/ ICH-GCP) 43](#__RefHeading___Toc154297854)

[8.3 Dokumentation unerwünschter Ereignisse (UE) und schwerwiegender unerwünschter Ereignisse (SUE) 43](#__RefHeading___Toc154297855)

[8.4 Meldung eines SUE durch den Prüfarzt 45](#__RefHeading___Toc154297856)

[8.5 Meldung schwerwiegender unerwarteter Nebenwirkungen (SUSAR- suspected unexpected serious adverse reaction) durch die WALA Heilmittel GmbH 45](#__RefHeading___Toc154297857)

[8.6 Vorzeitiger Abbruch der Studie in einem Zentrum 45](#__RefHeading___Toc154297858)

[8.7 Abbruch der gesamten klinischen Studie 45](#__RefHeading___Toc154297859)

[8.8 Safety Board 46](#__RefHeading___Toc154297860)

[9. Datenmanagement 46](#__RefHeading___Toc154297861)

[10. Statistische Methoden und Bestimmung der Fallzahl 48](#__RefHeading___Toc154297862)

[10.1 Biometrische Planung und Auswertung 48](#__RefHeading___Toc154297863)

[10.2 Biometrische Definition der Zielgrößen 48](#__RefHeading___Toc154297864)

[10.3 Fallzahlschätzung 49](#__RefHeading___Toc154297865)

[11. Studienmanagement 49](#__RefHeading___Toc154297866)

[11.1 Monitoring 49](#__RefHeading___Toc154297867)

[11.2 Audit / Inspektion 51](#__RefHeading___Toc154297868)

[11.3 Training des Studienpersonals 51](#__RefHeading___Toc154297869)

[11.4 Amendments 51](#__RefHeading___Toc154297870)

[12. Ethik / Bundesoberbehörde 51](#__RefHeading___Toc154297871)

[12.1 Ethische Durchführung der klinischen Prüfung 51](#__RefHeading___Toc154297872)

[12.2 Genehmigung durch das BfArM 51](#__RefHeading___Toc154297873)

[12.3 Bewertung durch die Ethikkommission 52](#__RefHeading___Toc154297874)

[12.4 Anzeige der klinischen Prüfung 52](#__RefHeading___Toc154297875)

[12.5 Patienteninformation und Einwilligungserklärung 52](#__RefHeading___Toc154297876)

[12.6 Datenschutz 52](#__RefHeading___Toc154297877)

[12.7 Versicherung 53](#__RefHeading___Toc154297878)

[12.8 Finanzielle Angelegenheiten 53](#__RefHeading___Toc154297879)

[12.9 Studienabschlussbericht / Publikation 53](#__RefHeading___Toc154297880)

[13. Vorgehensweise bei Notfall, Überdosierung oder Schwangerschaft 54](#__RefHeading___Toc154297881)

[13.1 Notfall 54](#__RefHeading___Toc154297882)

[13.2 Überdosierung, Schwangerschaft 54](#__RefHeading___Toc154297883)

[14. Literatur 55](#__RefHeading___Toc154297884)

[15. Anhänge 57](#__RefHeading___Toc154297885)

SYNOPSIS

|  |
| --- |
|  |
| **Prospektive, randomisierte, kontrollierte, multizentrische, teils doppelblinde Studie zum Vergleich von Disci/Rhus toxicodendron comp.®, Placebo und Warteliste bei Patienten mit chronischen LWS-Schmerzen**  Kurztitel: DISCI-Studie |
|  |

1. Leiter der klinischen Prüfung

PD Dr. med. Claudia M. Witt, MBA

Institut für Sozialmedizin, Epidemiologie und Gesundheitsökonomie

Charité Universitätsmedizin Berlin (im folgenden Studienzentrale genannt)

10098 Berlin

Tel: ++49 (30) 450529002; Fax: ++49 (30) 450529917

[claudia.witt@charite.de](mailto:claudia.witt@charite.de)

2. Studienzentren und vorgesehene Patientenzahl

An der Durchführung dieser Studie werden sich etwa 10-15 Prüfzentren (Kliniken/Praxen) beteiligen. Damit 123 Patienten, die eine mindestens 8-wöchige Therapie durchlaufen haben, auswertbar sind, müssen 150 Patienten randomisiert werden.

Die Patienten werden in die folgenden Behandlungsarme randomisiert: Verumgruppe, Placebogruppe und Wartelistengruppe.

Vorgesehen ist, zwischen 5 und 50 Patienten pro Zentrum zu randomisieren.

3. Zeitlicher Rahmen

| **Voraussichtlicher/ Geplanter zeitlicher Rahmen der Studie** | **Datum** |
| --- | --- |
| Vorbereitungsphase (4 Monate)  (Erstellung der kompletten Studienunterlagen, Rekrutierung der Prüfzentren, Einholung der Ethikvoten, Prüfarztschulung, Genehmigung BfArM) | bis 28.02.2007 |
| Patientenrekrutierungsphase (26 Wochen) | 1.03.2007 – 31.08.2007 |
| Voraussichtlicher Zeitpunkt der Aufnahme des ersten Patienten | 1.03.2007 |
| Behandlungsphase (8 Wochen pro Patient) | 1.03.2007 – 31.10.2007 |
| Follow-up Phase (18 Wochen pro Patient) | 1.05.2007 – 29.02.2008 |
| Voraussichtlicher Abschlusszeitpunkt des letzten Patienten | 29.02.2008 |
| Clean File | 1.03.2008 – 30.04.2008 |
| Auswertungsphase  (Auswertung, Erstellung des Berichts) | 1.05.2008 – 30.06.2008 |
| Studienende | 30.06.2008 |

4. Ziele

**Primäres Ziel**

Primäres Ziel ist zu untersuchen, ob Disci/Rhus toxicodendron. comp.® bei der Behandlung von chronischen Schmerzen der Lendenwirbelsäule wirksamer ist als keine Behandlung (Wartelistengruppe) bzw. wirksamer ist als ein Placebo.

**Sekundäre Ziele**

Sekundäre Ziele sind zu untersuchen, ob Disci/Rhus toxicodendron comp.® bezüglich Rückenfunktion (Funktionsfragebogen Hannover – Rücken FFbH-R^1^), Anzahl von Tagen mit Akutmedikationsbedarf in den Wochen 4-8 (Patiententagebuch), Anzahl der Arbeitsunfähigkeitstage bei erwerbstätigen Studienteilnehmern, Gesundheitsbezogene Lebensqualität (SF-36^2^), Emotionale Schmerzbewertung (Schmerzempfindungsskala SES^3^), Beurteilung der Beeinträchtigung im Alltag (Pain Disability Index^4^), Einfluss der Erwartungshaltung des Patienten auf den Outcome, Einfluss der Erwartungshaltung des Arztes auf den Outcome wirksamer ist als keine Behandlung (Wartleistengruppe) bzw. wirksamer ist als eine Placebobehandlung.

Zusätzlich erfolgt ein explorativer Vergleich zwischen Placebo- und Wartelistengruppe für die primären und sekundären Zielgrößen.

**5. Studiendesign**

Prospektive, randomisierte, kontrollierte, teils doppelblinde (Verum- vs. Placebo), teils offene (Vergleich mit Wartelistengruppe), multizentrische Studie zum Vergleich der Wirksamkeit von Disci/Rhus toxicodendron comp.® vs. unbehandelte Kontrollgruppe (Wartelistengruppe) und vs. Placebo.

6. Ziel-Patientenpopulation

**Einschlusskriterien**

In die Studie werden insgesamt 150 Patienten aufgenommen, auf die folgende Einschlusskriterien

zutreffen:

1. Männliche und/ oder weibliche Studienteilnehmer im Alter zwischen 30 und 75 Jahren
2. Bereitschaft des Studienteilnehmers, den Anweisungen des Prüfarztes zu folgen, d.h. die Studienbedingungen einzuhalten
3. Vorliegen der klinischen Diagnose „chronische Schmerzen der Lendenwirbelsäule“ (LWS) (chronische lokale oder pseudoradikuläre Schmerzen im Bereich der LWS aufgrund z.B. einer degenerativen Grunderkrankung mit Ausschluss der unten genannten Diagnosen)
4. Schmerzen der LWS seit mindestens 12 Monaten (= chronische LWS-Schmerzen), die therapeutischen Standardtherapiemaßnahmen müssen ausgeschöpft sein
5. Durchschnittswert der Schmerzintensität bezogen auf die letzten 7 Tage ≥ 40 mm auf einer visuellen Analogskala von 0-100 mm
6. In den letzten 4 Wochen nur medikamentöse Therapie der LWS-Beschwerden (Bedarfsbehandlung mit peripher wirkenden Analgetika, nichtsteroidalen Antiphlogistika und Muskelrelaxantien)
7. Bereitschaft einer ausreichenden/effektiven Kontrazeption bei Frauen im gebärfähigen Alter. Es gelten kontrazeptive Maßnahmen als effektiv, wenn die Versagerate <1%/Jahr beträgt, z.B. orale hormonelle Verhütung mit Kombinationspräparaten, Implantate, Intrauterinpessar, Diaphragmapessar, Portioklappe oder sexuelle Abstinenz.
8. Schriftliche Einwilligungserklärung

**Ausschlusskriterien**

In die Studie werden insgesamt 150 Patienten aufgenommen, auf die folgende Ausschlusskriterien nicht zutreffen:

1. Bereits zuvor oder aktuell durchgeführte Behandlung mit Disci-Präparaten
2. Bedarfsbehandlung der Schmerzen mit anderen als peripher wirkenden Analgetika und nichtsteroidalen Antiphlogistika (NSAR)
3. Regelmäßige (> 1/Woche) Einnahme von Schmerzmitteln aufgrund von anderen Erkrankungen
4. Protrusion oder Prolaps einer/mehrerer Bandscheibe(n) mit neurologischer Symptomatik
5. Vorausgegangene Wirbelsäulenoperationen
6. V.a. infektiöse Spondylopathie
7. LWS-Beschwerden aufgrund einer malignen und entzündlichen Erkrankung
8. Organische Ursachen der Beschwerden wie z.B. Morbus Bechterew, Morbus Reiter oder Morbus Behçet
9. Angeborene Missbildungen der Wirbelsäule mit Ausnahme leichter Lordosen, Kyphosen oder Skoliosen
10. V.a. Osteoporose mit Kompressionsfraktur eines oder mehrerer Wirbelkörper
11. V.a. spinale Stenosen
12. Spondylolyse oder Spondylolisthesis
13. Physiotherapie in den letzten 4 Wochen vor Studienbeginn oder während der Studie geplant
14. Beginn einer neuen Therapie zur Behandlung der LWS-Schmerzen in den vergangenen 4 Wochen
15. Bereits geplante neue Therapie (inklusive Operationen) zur Behandlung der LWS-Schmerzen im Studienzeitraum
16. Komplementärmedizinische Therapie (z.B. Akupunktur, Homöopathie, Phytotherapie, Neuraltherapie) in den letzten 4 Wochen vor Studienbeginn oder während der Studie geplant
17. Patienten, bei denen davon auszugehen ist, dass sie aus sprachlichen, intellektuellen bzw. anderen Gründen nicht in der Lage sind, die Bedeutung der klinischen Studie zu erkennen und die dafür notwendige Compliance aufzubringen
18. Patienten mit Alkohol-, Medikamenten- und/oder Drogenabhängigkeit
19. Gleichzeitige Teilnahme an anderen klinischen Studien. Die letzte Teilnahme an einer klinischen Studie muss mindestens 3 Monate zurückliegen
20. Vorliegen einer schwerwiegenden und/oder akuten oder chronischen organischen oder psychischen Erkrankung mit starker Beeinträchtigung des Allgemeinbefindens, die eine regelmäßige Studienteilnahme nicht möglich machen
21. Patienten mit Gerinnungsstörungen oder unter Einnahme gerinnungshemmender Medikamente
22. Schwangere Patientinnen (Vorliegen eines positiven Schwangerschaftstests bei der Einschlussuntersuchung) und Stillende
23. Patienten mit laufendem Rentenantrag
24. Studienteilnehmer, die mit der Planung und Durchführung der Studie betraut sind
25. Bekannte Überempfindlichkeit gegen einen der Inhaltsstoffe

7. Prüfpräparate, Dosierung und Applikationsmodus

**Beschreibung und Zusammensetzung der Prüfpräparate:**

- **Verum-Gruppe und Wartelisten-Gruppe erhalten:**

| **Disci/Rhus toxicodendron comp.®** | | |
| --- | --- | --- |
| Darreichungsform | Flüssige Verdünnung zur Injektion | |
| 1 Ampulle zu 10 ml enthält: | | |
| Wirksame Bestandteile | Aconitum napellus e tubere ferm 33c Dil. D4  Argentum metallicum Dil. D18 aquos.  Arnica montana e planta tota ferm 33c Dil. D18  Disci intervertebrales bovis (cervicales, thoracici et lumbales) Gl Dil. D6  Formica rufa ex animale toto Gl Dil. D5  Gelsemium sempervirens e rhizoma ferm 35b Dil. D2  Granit Dil. D8  Leontopodium alpinum e planta tota ferm 36 Dil. D2  Mandragora officinarum e radice ferm 34d Dil. D4  Phyllostachys e nodo ferm 35c Dil. D4  Toxicodendron quercifolium e foliis ferm 33d Dil. D4 | 0,1 g  0,1 g  0,1 g  0,1 g  0,1 g  0,1 g  0,1 g  0,1 g  0,1 g  0,1 g  0,1 g |
| Sonstige Bestandteile | Natriumchlorid, Natriumhydrogencarbonat, Wasser für Injektionszwecke | |

- **Placebo-Gruppe erhält:**

| Isotone Lösung | |
| --- | --- |
| Darreichungsform | Flüssige Verdünnung zur Injektion |
| 1 Ampulle zu 10 ml enthält: | |
| Wirksame Bestandteile | keine |
| Sonstige Bestandteile | Natriumchlorid, Natriumhydrogencarbonat, Wasser für Injektionszwecke |

**Dosierung, Art und Dauer der Anwendung:**

Verumgruppe:

Die Patienten der Verumgruppe erhalten 12 Veruminjektionen: 8 in den ersten 4 Wochen (2x/Woche im Abstand von mindestens einem Tag ohne Therapie) und 4 in den letzten 4 Wochen (1x/Woche im Abstand von mindestens 3 Tagen ohne Therapie). Injiziert wird pro Behandlung eine 10 ml Ampulle in 5 bis 10 Teilmengen.

Placebogruppe:

Die Patienten der Placebogruppe erhalten 12 Placeboinjektionen: 8 in den ersten 4 Wochen (2x/Woche im Abstand von mindestens einem Tag ohne Therapie) und 4 in den letzten 4 Wochen (1x/Woche im Abstand von mindestens 3 Tagen ohne Therapie). Injiziert wird pro Behandlung eine 10 ml Ampulle in 5 bis 10 Teilmengen.

Wartelistengruppe:

Den Patienten der Wartelistengruppe wird nach Abschluss der Wartezeit eine Behandlung mit Disci/Rhus toxicodendron comp.® angeboten. Das angebotene Dosierungsschema entspricht dem der Verumgruppe.

8. Testdauer

Die Gesamtbeobachtungszeit pro Patient beträgt 26 Wochen. Die Dauer der Untersuchung setzt sich für die Patienten der Verum- und Placebogruppe aus der Behandlungsphase (8 Wochen) und der Follow up Phase (18 Wochen) zusammen. Die Dauer der Untersuchung beinhaltet für die Patienten der Wartelistengruppe eine 26-wöchige Wartephase.

Die voraussichtliche Gesamtdauer der Untersuchungen (voraussichtlicher Zeitpunkt der Aufnahme des ersten Patienten bis zum voraussichtlichen Abschluss des letzten Patienten) ist auf 12 Monate geplant.

9. Zielgrößen

**Primäre Zielgröße**

Primäre Zielgröße zur Erfassung der Wirksamkeit von Disci/Rhus toxicodendron comp.® sind die

durchschnittlichen Schmerzen bezogen auf die letzten 7 Tage (gemessen anhand einer VAS (0-100

mm))^5^ nach 8 Wochen.

**Sekundäre Zielgrößen**

Sekundäre Zielgrößen sind:

1. Rückenfunktion (Funktionsfragebogen Hannover – Rücken FFbH-R^1^) nach 8 und 26 Wochen
2. Durchschnittliche Schmerzen bezogen auf die letzten 7 Tage (visuelle Analogskala (0-100 mm))^5^ nach 26 Wochen
3. Anzahl von Tagen mit Akutmedikationsbedarf in den Wochen 4 bis 8 (Patiententagebuch)
4. Anzahl der Arbeitsunfähigkeitstage bei erwerbstätigen Studienteilnehmern in den Wochen 4-8 (Patiententagebuch)
5. Anzahl von Arztbesuchen aufgrund von Rückenschmerzen in den Wochen 4-8 (Patiententagebuch)
6. Gesundheitsbezogene Lebensqualität (SF-36^2^) nach 8 und 26 Wochen
7. Emotionale Schmerzbewertung (Schmerzempfindungsskala SES^3^) nach 8 und 26 Wochen
8. Beurteilung der Beeinträchtigung im Alltag (Pain Disability Index^4^) nach 8 und 26 Wochen
9. Einfluss der Erwartungshaltung des Patienten auf den Outcome nach 8 und 26 Wochen
10. Einfluss der Erwartungshaltung des Arztes auf den Outcome nach 8 und 26 Wochen
11. 36% Responderraten für die durchschnittlichen Schmerzen nach 8 und 26 Wochen bezogen auf die letzten 7 Tage (visuelle Analogskala 0-100 mm)^5^

10. Sicherheit

Es erfolgt die kontinuierliche Sammlung und Auswertung von unerwünschten Ereignissen (UE) und schwerwiegenden unerwünschten Ereignissen (SUE). Die Kausalität jedes UE / SUE wird von den Prüfärzten beurteilt. Die Kausalität jedes SUE wird von der Abteilung Pharmakovigilanz der WALA Heilmittel GmbH unabhängig geprüft und ggf. entsprechend kategorisiert (Verdachtsfall einer unerwarteten schwerwiegenden Nebenwirkung (suspected unexpected serious adverse reaction).

Ein Safety Board unterstützt die Gewährleistung der Sicherheit für die Studienpatienten.

11. Statistische Methoden

**Biometrische Planung und Auswertung**

Durch das Design der Studie ergeben sich in den Zielgrößen verschiedene Abhängigkeiten, die bei der statistischen Auswertung berücksichtigt werden müssen. Generell werden zum einen beim einzelnen Patienten wiederholte Messungen durchgeführt, zum zweiten werden Patienten, die aus derselben Praxis stammen, möglicherweise ähnlichere Werte aufweisen als Patienten aus verschiedenen Praxen. Die Auswertungen werden daher grundsätzlich mit repeated measurement multilevel models durchgeführt (d.h. Multilevelmodell mit Messwiederholungen wenn notwendig). Die unterste Ebene ist dabei die Wiederholung, die zweite Ebene der Patient und die oberste Ebene die Praxis. Für die unterste Ebene wird als Korrelationsstruktur ein AR(1)-Prozess unterstellt (autoregressiver Prozess der Ordnung 1). Für die zweite Ebene wird eine austauschbare (‚exchangeable’) Korrelationsstruktur angenommen. Therapiegruppe und Messzeitpunkt werden als Faktoren in die Modelle eingeführt.

Folgende Kovariaten werden für die Modellbildung, gegebenenfalls Untergruppenbildung a priori festgelegt:

Patientenebene: - der jeweilige Baselinewert der betrachteten Zielgröße (entspricht einer ANCOVA)

Praxisebene: - Zentrumsgröße (groß/klein, cutoff ist vor Datenbankschließung festzulegen)

- akademischer Status (universitär vs. nicht-universitär)

- CAM-Angebot (ja/nein)

**Biometrische Definition der Zielgrößen**

Die **primäre Analyse** besteht aus zwei getrennten Zwei-Gruppen-Vergleichen der Werte der Primärzielgröße zum Messzeitpunkt 8 Wochen.

1) Vergleich zwischen Verum- und Placebo-Gruppe

2) Vergleich zwischen Verum- und Wartelistengruppe

(jeweils unter Einschluss des Baselinewertes (ANCOVA)).

Die Nullhypothese für jeden der beiden Vergleiche lautet:

„Es besteht kein Unterschied zwischen den Erwartungswerten in beiden Gruppen“.

Die Alternativhypothese lautet jeweils:

„Es besteht ein Unterschied zwischen den Erwartungswerten in beiden Gruppen“.

Beide Vergleiche werden mit dem zugehörigen zweiseitigen Wald-Test zum Niveau 2,5% durchgeführt, um ein Gesamtniveau von 5% sicherzustellen. Fehlende Werte werden grundsätzlich nicht ersetzt. In einer nachfolgenden Sensitivitätsanalyse wird eine Maximum-Likelihood basierte Imputation fehlender Werte sowie eine Worst-Case-Analyse vorgenommen, um die Belastbarkeit der Ergebnisse zu untersuchen.

Für die **sekundäre Analyse** werden zusätzlich die oben genannten Praxisfaktoren ins Modell

eingeschlossen. Ferner erfolgt ein Vergleich von Placebo- und Wartelistengruppe sowie Vergleiche

der 26-Wochen-Werte ohne weitere multiple Anpassung des Testniveaus. Die Analysen der

sekundären Endpunkte erfolgen analog.

Zusätzlich zur beschriebenen prospektiv geplanten konfirmatorischen Analyse werden explorativ

weitere Kovariaten und Modifier-Variablen in die Modelle eingeführt, um Hypothesen für weitere

Forschungsarbeiten zu gewinnen.

**Fallzahlschätzung**

Wenn 50 Patienten pro Gruppe eingeschlossen werden und durch eine angenommene Drop-out-Rate von 18% nach 8 Wochen noch 41 Patienten pro Gruppe für die Auswertung zu Verfügung stehen, so lässt sich ein Unterschied von 12,5 Einheiten auf der VAS und einer Standardabweichung von 18 Einheiten (Effektgröße 0,69) mit einer Power von 80% bei einem Testniveau von 5% statistisch nachweisen.

Flow Chart für den Arzt

| **Visiten**  **Prozeduren** | **Einschluss =**  **Visit 1** | **Visite 2 - 8** | | | | | | | | | | | | | | | **Visite 9 - 12** | | | | | | | **(keine**  **Visite)** | | **Verum-, Placebo-,**  **Wartelistengruppe**  **siehe Kapitel** | |
| --- | --- | --- | --- | --- | --- | --- | --- | --- | --- | --- | --- | --- | --- | --- | --- | --- | --- | --- | --- | --- | --- | --- | --- | --- | --- | --- | --- |
| Woche | **1** | **1-4** | | | | | | | | | | | | | | | **5-8** | | | | | | | **26** | |  | |
| **Alle Patienten** |  |  | | | | | | | | | | | | | | |  | | | | | | |  | |  | |
| Patienteninformation/ Einwilligung | **x** |  | |  | |  | |  | |  | |  | |  | | |  |  | |  | |  | |  | | **7.2.2** | |
| Schmerzen auf der VAS ≥ 40 mm | **x** |  | |  | |  | |  | |  | |  | |  | | |  |  | |  | |  | |  | | **7.2.3** | |
| Anamnese | **x** |  | |  | |  | |  | |  | |  | |  | | |  |  | |  | |  | |  | | **7.2.3** | |
| Körperliche Untersuchung | **x** |  | |  | |  | |  | |  | |  | |  | | |  |  | |  | |  | |  | | **7.2.4** | |
| Schwangerschaftstest | **x** |  | |  | |  | |  | |  | |  | |  | | |  |  | |  | |  | |  | | **7.2.3** | |
| Ein-/ Ausschlusskriterien | **x** |  | |  | |  | |  | |  | |  | |  | | |  |  | |  | |  | |  | | **7.2.4** | |
| Soziodemographische Daten | **x** |  | |  | |  | |  | |  | |  | |  | | |  |  | |  | |  | |  | | **7.2.4** | |
| Dokumentation der Begleitmedikation | **x** |  | |  | |  | |  | |  | |  | |  | | |  |  | |  | |  | |  | | **5.2** | |
| Dokumentation der Begleitbehandlung | **x** |  | |  | |  | |  | |  | |  | |  | | |  |  | |  | |  | |  | | **5.2** | |
| Fragen zur Erwartungshaltung | **x** |  | |  | |  | |  | |  | |  | |  | | |  |  | |  | |  | |  | | **6.1.6** | |
| Randomisierung | **x** |  | |  | |  | |  | |  | |  | |  | | |  |  | |  | |  | |  | | **7.2.6** | |
| **Verum- und Placebogruppe** |  |  | |  | |  | |  | |  | |  | |  | | |  |  | |  | |  | |  | |  | |
| Kurzanamnese |  | **x** | | **x** | | **x** | | **x** | | **x** | | **x** | | **x** | | | **x** | **x** | | **x** | | **x** | |  | | **7.3.1** | |
| Orientierende körperliche Untersuchung |  |  | |  | |  | |  | |  | |  | |  | | |  |  | |  | | **x** | |  | | **7.3.1** | |
| Injektion | **x** | **x** | | **x** | | **x** | | **x** | | **x** | | **x** | | **x** | | | **x** | **x** | | **x** | | **x** | |  | | **6.7/7.2.6** | |
| Drug Accountability | **x** | **x** | | **x** | | **x** | | **x** | | **x** | | **x** | | **x** | | | **x** | **x** | | **x** | | **x** | |  | | **5.1.8** | |
| Änderung der Begleitmedikation |  | **x** | | **x** | | **x** | | **x** | | **x** | | **x** | | **x** | | | **x** | **x** | | **x** | | **x** | |  | | **5.2/7.3.1** | |
| Änderung der Begleitbehandlung |  | **x** | | **x** | | **x** | | **x** | | **x** | | **x** | | **x** | | | **x** | **x** | | **x** | | **x** | |  | | **5.2/7.3.1** | |
| UE/ SUE | **x** | **x** | | **x** | | **x** | | **x** | | **x** | | **x** | | **x** | | | **x** | **x** | | **x** | | **x** | | **x** | | **6.13/6.14** | |
| Therapiedokumentation | **x** | **x** | | **x** | | **x** | | **x** | | **x** | | **x** | | **x** | | | **x** | **x** | | **x** | | **x** | |  | | **6.7** | |
| Patientenfragebogen 1 ausgeben | **x** |  | |  | |  | |  | |  | |  | |  | | |  |  | |  | |  | |  | | **7.2.5** | |
| Patiententagebuch 1 ausgeben | **x** |  | |  | |  | |  | |  | |  | |  | | |  |  | |  | |  | |  | | **7.2.7** | |
| Befragung nach der 3. Behandlung |  |  | | **x** | |  | |  | |  | |  | |  | | |  |  | |  | |  | |  | | **6.8** | |
| Fragen zu Erfolg Verblindung |  |  | |  | |  | |  | |  | |  | |  | | |  |  | |  | | **x** | |  | | **7.3.1** | |
| Einschätzung der Wirksamkeit |  |  | |  | |  | |  | |  | |  | |  | | |  |  | |  | | **x** | |  | | **7.3.1** | |
| **Wartelistengruppe** |  |  | |  | |  | |  | |  | |  | |  | | |  |  | |  | |  | |  | |  | |
| Patientenfragebogen 1 ausgeben | **x** |  | |  | |  | |  | |  | |  | |  | | |  |  | |  | |  | |  | | **7.2.5** | |
| Patiententagebuch 1 ausgeben | **x** |  | |  | |  | |  | |  | |  | |  | | |  |  | |  | |  | |  | | **7.2.7** | |
|  | | |  | |  | |  | |  | |  | |  | |  |  | |  |  | |  | |  | |  | |  |

Liste der Abkürzungen und Definitionen

Die folgenden allgemeinen und fachspezifischen Abkürzungen werden im Studienprotokoll verwendet.

| **Allgemeine und fachspezifische Abkürzungen** | **Beschreibung** |
| --- | --- |
|  |  |
| Comp. | Compositorium |
| CRF | Case Report Form (Datenerhebungsbogen) |
| Dil. | Dilution |
| Disci / DISCI | Disci/Rhus toxicodendron comp.® |
| FFbH-R | Funktionsfragebogen Hannover-Rücken |
| GCP | Good Clinical Practice (Gute Klinische Praxis) |
| GMP | Good Manufacturing Practice (Gute Herstellungpraxis) |
| ICH | International Conference on Harmonisation |
| LKP | Leiter der Klinischen Prüfung |
| LWS | Lendenwirbelsäule |
| NSAR | Nichtsteroidale Antiphlogistika |
| PDI | Pain Disability Index |
| s.c. | subcutan |
| SES | Schmerzempfindungsskala |
| SF-36 | Lebensqualitätsfragebogen SF-36 |
| SOP | Standard Operating Procedure |
| SUE | Schwerwiegendes unerwünschtes Ereignis |
| UE | Unerwünschtes Ereignis |
| VAS | Visuelle Analogskala |

Liste Der Wichtigsten Formulare

| **Formular** | Erläuterung |
| --- | --- |
| Patienteninformation | Patient wird nach Kontaktaufnahme schriftlich über Wesen, Zweck, Behandlung, Bedeutung, mögliche Risiken und Nutzen sowie freiwillige Teilnahme an der klinischen Studie aufgeklärt |
| Pre-Study Screening Log | Jeder interessierte Patient wird in die Pre-Study Screening Log Liste mit folgenden Daten aufgenommen: laufende Screeningnummer, aktuelles Datum, Angaben zur Person, Aufnahme in die Studie ja/nein, eventueller Grund der Nichtaufnahme |
| Einwilligungserklärung | Der Studienteilnehmer sowie der Prüfarzt müssen die Einwilligungserklärung vor Beginn der studienspezifischen Untersuchungen eigenhändig datieren und unterschreiben |
| Faxmeldung „Patienteneinschluss“ | Wird innerhalb von 24 Stunden nach Einschluss eines neuen Patienten vom Prüfarzt an die Studienzentrale gefaxt.  Enthält Einschlussdatum, Patientencode und Name des Prüfarztes, Interventions- oder Wartelistengruppe |
| Patientenidentifikation | Wird innerhalb von 24 Stunden nach Einschluss eines neuen Patienten zusammen mit der Einwilligungserklärung vom Prüfarzt an die Studienzentrale per Post zugesandt.  Enthält: Einschlussdatum, Patientencode, Name und Adresse des Patienten, Name des Prüfarztes |
| Einschlussuntersuchung | Dokumentation der anamnestischen und Untersuchungsdaten bei der ersten Visite (Einschlussuntersuchung) des Patienten durch den Prüfarzt inklusive Checkliste Ein- und Ausschlusskriterien |
| Patiententagebuch 1-3 | Patient dokumentiert die Tage mit LWS-Schmerzen, was er dagegen unternommen hat, die Zahl der Arbeitsunfähigkeitstage und die Anzahl der Arztbesuche |
| Patientenfragebogen 1-3 | Erhebung der primären und sekundären Zielparameter, werden vom Patienten ausgefüllt |
| Therapiedokumentationen 1-12 | Dokumentation der einzelnen Behandlungen und Abschlussbewertung durch den Prüfarzt |
| Befragung nach der 3. Behandlung | Erhebt die Glaubwürdigkeit der Behandlung, wird jeweils von Arzt und Patient ausgefüllt |
| Randomisierungsumschläge | Enthalten die Randomisierungsnummer und die Mitteilung, ob der Patient in der Wartelisten- oder in den Interventionsgruppen ist |
| Notfallumschläge/Entblindungsformular | Enthalten die Gruppenzuordnung zur Randomisierungsnummer |
| Bericht über SUE | Bericht und Bewertung eines SUE durch den Prüfarzt |
| Bericht über UE | Dokumentation der UE durch den Prüfarzt |
| Therapie- und Studienabbruch | Dokumentation von Studienabbruch/ Therapieabbruch durch den Prüfarzt |
| Drug Accountability | Dokumentation der Ausgabe und Rücknahme der Prüfmedikation durch den Prüfarzt |
| Schwangerschaft Outcome-Bericht | Dokumentation einer Schwangerschaft, die während der Studie auftritt |
| Überdosierungsbericht | Dokumentation einer Überdosierung durch den Prüfarzt |

Nomenklatur im Prüfplan

| **Nomenklatur nach GCP** | **Nomenklatur im Prüfplan** |
| --- | --- |
| Hauptprüfer/Prüfer | Prüfarzt |
| Klinische Prüfung gemäß GCP | Studie |
| Prüfpräparat | Disci/Rhus toxicodendron comp.® oder Placebo |

PRÜFÄRZTE UND VERANTWORTLICHE

Leiter der klinischen Prüfung

(LKP) und Projektleitung Institut PD Dr. Claudia M. Witt, MBA

Institut für Sozialmedizin, Epidemiologie und

Gesundheitsökonomie

Charité – Universitätsmedizin Berlin

10098 Berlin

Tel: ++49 (30) 450529002; Fax: ++49 (30) 450529917

Email: [claudia.witt@charite.de](mailto:claudia.witt@charite.de)

Projektkoordinator Institut Dr. Benno Brinkhaus

Internist, Arzt für Naturheilverfahren und Akupunktur

Institut für Sozialmedizin, Epidemiologie und

Gesundheitsökonomie

Charité – Universitätsmedizin Berlin

10098 Berlin

Tel: ++49 (30) 450529079; Fax: ++49 (30) 450529917

Email: benno.brinkhaus@charite.de

Wissenschaftlicher Mitarbeiter

Institut N.N.

Sponsor (nach ICH-GCP) WALA Heilmittel GmbH

Dorfstr. 1

D-73087 Bad Boll / Eckwälden

Projektleiter WALA WALA Heilmittel GmbH

Dr. Ulrich Meyer

Ressortleiter Wissenschaft

Dorfstr. 1

D-73087 Bad Boll / Eckwälden

Email: [Ulrich.Meyer@wala.de](mailto:Ulrich.Meyer@wala.de)

Stufenplanbeauftragter

(Meldung SUEs) Peter Vögele

WALA Heilmittel GmbH

Dorfstr. 1

D-73087 Bad Boll / Eckwälden

Email: Peter.Voegele@wala.de

Tel. : ++ 49 (7164) 930 311; Fax. : ++49 (7164) 930 6989

Studienkoordination WALA/

Koordination Monitoring Anna Fischer

WALA Heilmittel GmbH

Dorfstr. 1

D-73087 Bad Boll / Eckwälden

Tel.: ++ 49 (7164) 930-0

Biometrie Prof. Dr. Karl Wegscheider

Institut für Statistik und Ökonometrie

Universität Hamburg

Von-Melle-Park 5

20146 Hamburg

Tel: +49 (40) 428 38 - 6451; Fax: +49 (40) 428 38 - 6236

Email: karl.wegscheider@t-online.de

Dipl. Stat. Stephanie Roll

Institut für Sozialmedizin, Epidemiologie und

Gesundheitsökonomie

Charité – Universitätsmedizin Berlin

10098 Berlin

Tel: ++49 (30) 450529023; Fax: ++49 (30) 450529917

Email: stephanie.roll@charite.de

Studienzentrale Beatrice Eden

Institut für Sozialmedizin, Epidemiologie und

Gesundheitsökonomie

Charité – Universitätsmedizin Berlin

10098 Berlin

Tel: ++49 (30) 450529027; Fax: ++49 (30) 450529917

Email: [beatrice.eden@charite.de](mailto:beatrice.eden@charite.de)

Iris Bartsch

Institut für Sozialmedizin, Epidemiologie und

Gesundheitsökonomie

Charité – Universitätsmedizin Berlin

10098 Berlin

Tel: ++49 (30) 450529027; Fax: ++49 (30) 450529917

Email: [iris.bartsch@charite.de](mailto:iris.bartsch@charite.de)

Datenmanagement Katja Wruck

Institut für Sozialmedizin, Epidemiologie und

Gesundheitsökonomie

Charité – Universitätsmedizin Berlin

10098 Berlin

Tel: ++49 (30) 450529076; Fax: ++49 (30) 450529917

Email: katja.wruck@charite.de

Ethikkommissionen

Landesamt für Gesundheit und Soziales

Geschäftsstelle der Ethikkommission des Landes Berlin

Sächsische Strasse 28

10707 Berlin

Tel: ++49 (30) 9012-7640; Fax: ++49 (30) 9012-7634

Email: [ethik-kommission@lageso.verwalt-berlin.de](mailto:ethik-kommission@lageso.verwalt-berlin.de)

Medizinische Fakultät der Universität Duisburg-Essen

Institut für Pharmakologie

Universitätsklinikum Essen

Hufelandstr. 55

45122 Essen

Tel: ++49 (201) 723-3113; Fax: ++49 (201) 723-5837

Email: [ethikkommission@uk-essen.de](mailto:ethikkommission@uk-essen.de)

Bayerische Landesärztekammer

Ethikkommission

Mühlbaurstr. 16

81677 München

Tel: ++49 (89) 41 47-212; Fax: ++ 49 (89) 41 47-334

Email: [ethikkommission@blaek.de](mailto:ethikkommission@blaek.de)

Landesärztekammer Brandenburg

Hauptgeschäftsstelle

Dreifertstr. 12

03044 Cottbus

Tel: ++ 49 (355) 78 01 00; Fax: ++ 49 (355) 7 80 10 36

Email: [post@laekb.de](mailto:post@laekb.de)

Landesärztekammer Thüringen

Ethikkommission

Im Semmicht 33

07751 Jena

Tel: ++49 (3641) 61 42 01; Fax: ++49 (3641) 61 42 09

Email: [ethikkomission@laek-thueringen.de](mailto:ethikkomission@laek-thueringen.de)

Ärztekammer Nordrhein

Ethikkommission

Tersteegenstr. 31

40474 Düsseldorf

Tel: ++ 49 (211) 4302-11581; Fax: ++ 49 (211) 4302-11585

Email: aerztekammer@aekno.de

Ärztekammer Westfalen-Lippe

Ethikkommission

Von-Esmarck-Str. 62

48149 Münster

Tel: ++ 49 (251) 8355290; Fax: ++ 49 (251) 83557097

Safety Board

Alexander von Humboldt Klinik

Dr. Johannes Wilkens

Dr. Gebhardt-Steuer-Str. 24

95138 Bad Steben

Tel: ++ 49 (9288) 920-400; Fax: ++49 (9288) 920-108

Email: [info@humboldtklinik.de](mailto:info@humboldtklinik.de)

Karl- und Veronica Carstens Stiftung

Im Stifterverband für die Deutsche Wirtschaft

Dípl. Stat. Rainer Lüdtke

Am Deimelsberg 36

45276 Essen

Tel. ++ 49 (201) 56305-16

Email: [r.luedtke@carstens-stiftung.de](mailto:r.luedtke@carstens-stiftung.de)

Kliniken Essen Mitte

Knappschaftskrankenhaus Innere V

PD Dr. Andreas Michalsen

Am Deimelsberg 34a

45276 Essen

Tel: ++49 (201) 805 4017; Fax: ++ 49 (201) 805 4005

Email: andreas.michalsen@uni-essen.de

Prüfzentren

Kliniken Essen Mitte

Knappschaftskrankenhaus, Innere V

Prof. Dr. med. Gustav Dobos

Am Deimelsberg 34a

45276 Essen

Tel: ++49 (201) 805 4017; Fax: ++ 49 (201) 805 4005

Email: gustav.dobos@uni-essen.de

MVZ Polikum Friedenau

Geschäftsführer Dr. Wolfram Otto und Dr. Susanne Schwarz

Rubensstraße 119

12157 Berlin

Tel: ++ 49 (30) 720 110 00, Fax: ++ 49 (30) 72 01 10 99

Email: arzt@friedenau.polikum.de

Institut für Sozialmedizin, Epidemiologie und Gesundheitsökonomie

Charité – Universitätsmedizin

Prof. Dr. Stefan N. Willich

Luisenstraße 57

10117 Berlin

Tel: ++49 (30) 450529002; Fax: ++49 (30) 450529917

Email: stefan.willich@charite.de

Dr. Sigrun Kokott

Uhlandstraße 53

03050 Cottbus

Tel: ++49 (355) 541836

Dr. Uta Stiegler

Kurfürstendamm 139

10711 Berlin

Tel: ++49 (30) 8929059; Fax: ++49 (30) 89090845

Email: praxis@praxis-stiegler.de

Dr. Jürgen Bachmann

August-Bebel-Straße 8-10

45525 Hattlingen

Tel: ++ 49 (2324) 92590; Fax: ++ 49 (2324) 925999

Dr. Ralf Birnbaum

Siebenmorgen 54

51427 Bergisch-Gladbach

Tel: ++ 49 (2204) 67368; Fax: ++ 49 (2204) 249429

Klinik für Innere Medizin II/ Kompetenzzentrum Naturheilverfahren

Prof. Dr. med. Christine Uhlemann

Bachstraße 18

07740 Jena

Tel: ++ 49 (3641) 933 180; Fax: ++49 (3641) 933 182

Email: christine.uhlemann@med.uni-jena.de

Dr. Konrad Beyer

Mühlenweg 4c

04924 Dobra

Tel: ++ 49 (35341) 901160 o. 901159; Fax: ++ 49 (35341) 10642

Email: Beyer-Dobra@t.online.de

Dr. Karin Fleßa

Altstadt 16

95028 Hof

Tel: ++ 49 (9281) 72550; Fax: ++ 49 (9281) 725519

Dr. Christine Dühn

Pestalozzistr. 10

03226 Vetschau

Tel: ++49 (035433) 4173; Fax: ++49 (035433) 72144

# Einleitung

## Allgemeiner Hintergrund

Schmerzen der Lendenwirbelsäule (LWS) sind ein bedeutendes Gesundheitsproblem in den industrialisierten Ländern mit gravierenden gesundheitsökonomischen Folgen^6^. Schmerzen der LWS sind definiert als Schmerzen, Muskelverhärtung oder Steifigkeit, die unterhalb des Rippenbogens und oberhalb der unteren Glutealfalte am Rücken lokalisiert sind, mit ggf. Schmerzausstrahlung in die Beine^6^. Auch wenn 90% der Patienten mit akuten LWS-Schmerzen innerhalb von 6 Wochen zur Beschwerdefreiheit kommen, entwickeln bis zu 7% der Patienten einen chronischen Schmerz^7^. Die häufigsten Symptome von unspezifischen Rückenschmerzen sind Schmerz und Arbeitsunfähigkeit. Die Lebenszeit-Prävalenz variiert zwischen 49% und 70% und die Punkt-Prävalenz zwischen 12% und 30%^7-9^. Erhebungen in Großbritannien zeigten, dass 16% aller Erwachsenen innerhalb eines Jahres ihren Arzt mindestens einmal wegen Rückenschmerzen konsultierten, wobei umgerechnet Kosten in Höhe von ca. 0,6 Mrd. Euro entstanden^10^.

Die Diagnostik und Behandlung von chronischen LWS-Schmerzen variiert innerhalb der Länder bzw. zwischen den Ländern unter Allgemeinärzten, Orthopäden und anderen Ärzten und Therapeuten^11,12^. Mehr als 1000 randomisierte kontrollierte Studien, die die Wirksamkeit von konventioneller und komplementärmedizinischer Therapie bei Patienten mit Schmerzen der LWS evaluieren, wurden publiziert^6^. Die Evidenz zur Behandlung von chronischem Rückenschmerz wurde in systematischen Reviews, u.a. einem Cochrane Review, zusammengefasst^13^. Die Effektivität vieler konventioneller Behandlungen konnte bisher nicht eindeutig belegt werden^14^. Konventionelle Therapieverfahren, für die es eine starke Evidenz zu einer Wirksamkeit gibt, sind Bewegungsübungen und intensivierte multidisziplinäre Schmerzbehandlungsprogramme. Dagegen ist die Evidenz für die Wirksamkeit von Verhaltenstherapien, Schmerzmitteln, Antidepressiva, nicht-steroidalen Antiphlogistika, Rückenschulprogrammen und Manueller Therapie nicht eindeutig belegt^13^ und die Wirksamkeit ist zeitlich begrenzt. Für die meisten konventionellen Therapieverfahren konnten keine relevanten Langzeiteffekte nachgewiesen werden^13^.

In Übersichtsarbeiten wird darauf hingewiesen, dass die Inanspruchnahme komplementärmedizinischer Verfahren durch Schmerzpatienten zunehmend ist^15-17^. Die Evidenz von komplementärmedizinischen Therapieverfahren deutet auf die Wirksamkeit von folgenden Verfahren bei LWS-Schmerzen hin: Massage, Spinal Manipulation, Akupunktur und Neuroreflexotherapie^18-20^.

## Hintergrund der klinischen Prüfung und Fragestellung

Disci/Rhus toxicodendron comp.® ist ein anthroposophisches Arzneimittel, das seit Jahrzehnten bei Wirbelsäulenerkrankungen eingesetzt wird. Eine Hauptindikation sind akute Schmerzzustände bei degenerativen Veränderungen der Wirbelsäule. Erfahrungsberichte aus der Praxis weisen darauf hin, dass die Applikation von 10 ml Disci/Rhus toxicodendron comp.® bei einem akuten Schmerzereignis über einige Tage zur deutlichen Schmerzlinderung, oftmals schon unmittelbar nach der Injektion, führt.

Weitere Erfahrungsberichte aus der therapeutischen Praxis geben den Hinweis, dass Disci/Rhus toxicodendron comp.®, verabreicht über einen längeren Zeitraum, auch bei Patienten mit chronischen Rückenbeschwerden eine Schmerzlinderung bewirken könnte. Über den Einsatz von Disci/Rhus toxicodendron comp.® bei chronischen Rückenschmerzen gibt es aber bislang keine systematisch erhobenen Daten, mit denen verallgemeinernd seine therapeutische Wirkung bei dieser Anwendung beschrieben werden kann.

Die vorliegende Studie soll die Wirksamkeit von Disci/Rhus toxicodendron comp.® bei Patienten mit chronischen Schmerzen der LWS untersuchen, mit dem Ziel, Daten zur Beurteilung der Wirksamkeit und Sicherheit zu generieren.

Konkret wird in der Studie untersucht, ob und in welchem Ausmaß eine subcutane (s.c.) Injektion von Disci/Rhus toxicodendron comp.® bei der Behandlung von Patienten mit chronischen Schmerzen im Bereich der LWS a) wirksamer ist als keine Behandlung (Wartelistengruppe) und b) wirksamer ist als eine s.c. Placeboinjektion.

# Studienziele

## Primäres Ziel und primäre Zielgröße

Primäres Ziel ist zu untersuchen, ob Disci/Rhus toxicodendron comp.® bei der Behandlung von chronischen Schmerzen der LWS wirksamer ist als keine Behandlung (Wartelistengruppe) bzw. wirksamer ist als ein Placebo.

Primäre Zielgröße sind die durchschnittlichen Schmerzen bezogen auf die letzten 7 Tage (gemessen anhand einer visuellen Analogskala (0-100 mm))^5^ nach 8 Wochen.

Für die (konfirmatorische) statistische Prüfung wurden folgende Arbeitshypothesen formuliert:

Die visuelle Analogskala der Schmerzen nach der letzten Behandlung unterscheidet sich zwischen der Verum- und der Wartelistengruppe (Hypothese 1) bzw. zwischen der Verum- und der Placebogruppe (Hypothese 2) nicht.

## Sekundäre Ziele und sekundäre Zielgrößen

**Sekundäre Ziele**

Sekundäre Ziele sind zu untersuchen, ob Disci/Rhus toxicodendron comp.® bei den folgenden genannten Parametern wirksamer ist als keine Behandlung (Wartleistengruppe) bzw. wirksamer ist als eine Placebobehandlung.

**Sekundäre Zielgrößen**

Sekundäre Zielgrößen sind:

1. Rückenfunktion (Funktionsfragebogen Hannover – Rücken FFbH-R^1^) nach 8 und 26 Wochen
2. Durchschnittliche Schmerzen bezogen auf die letzten 7 Tage (visuelle Analogskala (0-100 mm))^5^ nach 26 Wochen
3. Anzahl von Tagen mit Akutmedikationsbedarf in den Wochen 4 bis 8 (Patiententagebuch)
4. Anzahl der Arbeitsunfähigkeitstage bei erwerbstätigen Studienteilnehmern in den Wochen 4-8 (Patiententagebuch)
5. Anzahl von Arztbesuchen aufgrund von Rückenschmerzen in den Wochen 4-8 (Patiententagebuch)
6. Gesundheitsbezogene Lebensqualität (SF-36^2^) nach 8 und 26 Wochen
7. Emotionale Schmerzbewertung (Schmerzempfindungsskala SES^3^) nach 8 und 26 Wochen
8. Beurteilung der Beeinträchtigung im Alltag (Pain Disability Index^4^) nach 8 und 26 Wochen
9. Einfluss der Erwartungshaltung des Patienten auf den Outcome nach 8 und 26 Wochen
10. Einfluss der Erwartungshaltung des Arztes auf den Outcome nach 8 und 26 Wochen
11. 36% Responderraten für die durchschnittlichen Schmerzen nach 8 und 26 Wochen bezogen auf die letzten 7 Tage (visuelle Analogskala 0-100 mm)^5^

Zusätzlich erfolgt ein explorativer Vergleich zwischen Placebo- und Wartelistengruppe für die primären

und sekundären Zielgrößen.

## Nutzen – Risiko-Bewertung der Studie

**Nutzen:** Aufgrund von Erfahrungen in der praktischen Anwendung wird erwartet, dass 10 ml Disci/Rhus toxicodendron comp.® s.c. an mehreren Stellen im Schmerzbereich der LWS verabreicht, schmerzlindernd auf chronische Wirbelsäulenbeschwerden wirkt, dass die Beweglichkeit der Wirbelsäule zunimmt und der Verbrauch von Schmerzmitteln gesenkt werden kann.

Unter der Annahme, dass sich dies bei der Evaluierung der Kurz- und Langwirksamkeit und der Sicherheit von Disci/Rhus toxicodendron comp.® bei chronischen LWS-Schmerzen bestätigt, entsteht für die Patienten der Verumgruppe und der Wartelistegruppe ein direkter Nutzen durch die Studienteilnahme (Patienten in der Verumgruppe während der Studienbehandlungsphase, Patienten in der Wartelistengruppe im Anschluss an die Studienbehandlungsphase).

Durch die insgesamt gewonnenen Erkenntnisse bzgl. Wirksamkeit und Sicherheit einer Behandlung mit 10 ml Disci/Rhus toxicodendron comp.® kann die zukünftige Behandlung von Patienten mit chronischen LWS-Beschwerden möglicherweise durch ein sehr gut verträgliches Medikament ergänzt werden, das insbesondere den Verbrauch von nebenwirkungsreichen Schmerzmitteln reduziert. Hieraus ergibt sich ein zukünftiger Nutzen für die Gesamtgruppe der Patienten mit chronischen LWS-Beschwerden und damit auch für die Studienteilnehmer der Placebogruppe.

**Risiken:** Disci/Rhus toxicodendron comp.® ist ein zugelassenes und sehr gut verträgliches Arzneimittel, das schon seit einigen Jahrzehnten auf dem europäischen Markt erhältlich ist. Die Wahrscheinlichkeit, dass bei bestimmungsgemäßem Gebrauch während der Studie Nebenwirkungen auftreten, ist im Hinblick auf die langjährige Erfahrung mit dem Arzneimittel auf dem Markt als sehr gering zu bewerten.

Bestandteile von Disci/Rhus toxicodendron comp.® können bei empfindlichen Patienten allergische Reaktionen auslösen. Um dieses Risiko so klein wie möglich zu halten, werden Patienten, bei denen eine Allergie gegen einen der Inhaltsstoffe von Disci/Rhus toxicodendron comp.® bekannt ist, von der Studienteilnahme ausgeschlossen. Nicht ausgeschlossen werden kann, wie bei jeder Anwendung eines zugelassenen Arzneimittels, dass eine bislang unbekannte Überempfindlichkeit bzw. eine unter der Behandlung auftretende Sensibilisierung zu einer allergischen Reaktion als Folge der Anwendung führen kann. Die Wahrscheinlichkeit des Neuauftretens einer allergischen Reaktion wird im Hinblick auf die langjährige Erfahrung mit dem Arzneimittel auf dem Markt und die bisher gute Verträglichkeit als sehr gering eingeschätzt. Darüber hinaus werden die Patienten im Rahmen der Sammlung von Sicherheitsdaten der Studie sehr ausführlich auf ihr Befinden hin befragt, so dass mögliche Unverträglichkeiten zeitnah erfasst werden können.

Nebenwirkungen durch die Verabreichung der Placebo-Lösung werden nicht erwartet.

Die Prüfpräparate werden s.c. appliziert. Dies kann für den Patienten leicht schmerzhaft sein. Das Auftreten von kleinen Hämatomen oder einer lokalen Infektion um die Einstichstellen herum lässt sich nicht völlig ausschließen. Die Injektionen werden von den Prüfärzten durchgeführt, die in der s.c. Applikation von Schmerzmitteln erfahren sind. Es wird davon ausgegangen, dass die Belastung durch die Injektion und das verbleibende Restrisiko gering sind.

**Risiko-Nutzen-Bewertung:** Bei der Durchführung der vorliegenden klinischen Studie wird für die teilnehmenden Patienten - wie oben ausgeführt - nicht mit wesentlichen Risiken oder Belastungen gerechnet.

Demgegenüber steht die Erwartung, dass chronische LWS-Beschwerden mit Disci/Rhus toxicodendron comp.® hinsichtlich Schmerzlinderung, verbesserter Beweglichkeit und insbesondere der Einsparung von nebenwirkungsreichen Schmerzmitteln erfolgreich behandelt werden können.

Da die Durchführung der Studie nicht mit großen Risiken verbunden ist und ein Nutzen durch die Behandlung mit Disci/Rhus toxicodendron comp.® erwartet wird, fällt die Risiko-Nutzen-Abwägung für die vorliegende Studie deutlich positiv aus. Aus ärztlicher Sicht sind die Durchführung der Studie und die Teilnahme an der Studie vertretbar.

# Studiendesign

Prospektive, randomisierte, kontrollierte, teils doppelblinde (Verum- vs. Placeboinjektion), teils offene (Vergleich mit Wartelistengruppe), multizentrische Studie zum Vergleich der Wirksamkeit von Disci/Rhus toxicodendron comp.® vs. unbehandelte Kontrollgruppe (Wartelistengruppe) bzw. vs. Placeboinjektion. Die primäre Zielgröße (die durchschnittlichen Schmerzen bezogen auf die letzten 7 Tage, gemessen auf der VAS 0-100 mm) wird nach 8 Wochen Behandlungsphase bestimmt. Die Gesamtbeobachtungsdauer pro Patient beträgt 26 Wochen.

Abbildung 1: Studiendesign

## Hintergründe zum Studiendesign, Dosis und Kontrollgruppe

Studiendesign:

Dieses Design wurde gewählt, um das Ausmaß des Gesamteffektes einer Injektion von Disci/Rhus toxicodendron comp.® zu untersuchen (Verumgruppe vs. Wartelistengruppe), gleichzeitig aber auch das Ausmaß des unspezifischen Effektes (Placeboeffekt) des Verums zu überprüfen (Verumgruppe vs. Placebogruppe). Die Länge der Gesamtbeobachtungsdauer von 26 Wochen wurde gewählt, um evtl. Langzeiteffekte beurteilen zu können. Aus ethischen und aus Motivationsgründen wird den Patienten der Wartelistengruppe nach 26 Wochen Wartezeit eine Behandlung mit Disci/Rhus toxicodendron comp.® angeboten. Diese Behandlung ist jedoch nicht mehr Teil der Evaluation.

Die Studienteilnahme des einzelnen Studienteilnehmers erstreckt sich vom Einschluss in die Studie (schriftliche Einwilligung zur Studienteilnahme) bis zur Abgabe des dritten Fragebogens (Woche 26 nach Randomisierung).

**Multizentrische Durchführung:**

Die Studie wird als multizentrische Studie an 10 – 15 Zentren durchgeführt. Es sollen mind. 5 bis max. 50 Patienten pro Zentrum (je nach Größe des Prüfzentrums) eingeschlossen werden.

Randomisierung und Verblindung:

Es wird einen Blockrandomisierung geschichtet nach Zentren durchgeführt. Für den Vergleich Verum vs. Placebo wird die Studie doppelblind durchgeführt (Patient, Arzt, Statistiker (bei der Auswertung der primären Zielgröße)), während für den Vergleich Verum vs. Warteliste die Studie offen ist. Mit der Randomisierung wird eine ausgeglichene Verteilung der Patientencharakteristika bei Baseline vor Studienintervention angestrebt und gleichzeitig eine optimalere Beurteilung der Studienergebnisse ermöglicht. Die Verblindung von Patient, Arzt und Statistiker erfolgt, um einen Bias (z.B. Erwartungshaltung zur Therapie) zu vermeiden.

Dosierung:

Die Dosierung und das Dosierungsschema von Disci/Rhus toxicodendron comp.® richtet sich nach der Gebrauchsinformation „Disci/Rhus toxicodendron comp.® 10 ml“, da es das Ziel der vorliegenden Studie ist, systematisch Daten zur Wirksamkeit und Sicherheit des Präparates mit der 10 ml Dosierung bei chronischen LWS-Beschwerden unter den Bedingungen der Praxis zu erheben.

## Randomisierung

Es wird eine Blockrandomisierung geschichtet nach Zentren vorgenommen. Das Zuteilungsverhältnis Verum : Placebo : Warteliste beträgt 1 : 1 : 1 (d.h. 50 : 50 : 50 Patienten). Die Randomisierungsliste wird mit der Software SAS von einem Statistiker, der nicht an der Auswertung des primären Zielparameters beteiligt ist, erstellt. Diese wird an den Prüfpräparatekoordinator der Gruppe Klinische Forschung/ Pharmakovigilanz der WALA GmbH übermittelt und zusätzlich in einem versiegelten Umschlag im Tresor der Studienzentrale verwahrt. Die Studienärzte erhalten Briefumschläge in aufsteigender Nummerierung, die eine zufällig generierte Randomisierungsnummer enthalten. Die Randomisierungnummer entspricht dem Patientencode und der Nummer der Studienmedikation für den jeweiligen Patienten. Weder dem Prüfarzt noch dem Patienten noch der Studienzentrale wird die Gruppe (auch nicht in verblindeter Form z.B. Gruppe 1 oder Gruppe 2) mitgeteilt.

# Auswahl der Studienpopulation

## Rekrutierung

Die Rekrutierung der 150 Patienten erfolgt direkt über die Prüfzentren (Kliniken/Praxen) aus dem normalen Patientenklientel. Es sind keine Anzeigen in Zeitungen geplant.

Wenn der Prüfarzt Patienten mit chronischen LWS-Schmerzen unter dem Patientenklientel hat, werden diese Patienten von ihm auf die klinische Prüfung aufmerksam gemacht. Patienten, die an einer Studienteilnahme interessiert sind, werden vom Prüfarzt ausführlich über die klinische Prüfung aufgeklärt. Patienten, die schriftlich in die Studienteilnahme einwilligen, werden bei vorliegender Eignung in die klinische Prüfung eingeschlossen und genau auf ihre Eignung untersucht. Bei nicht vorliegender Eignung endet die Studienteilnahme. Es wird eine Rekrutierungszeit von 6 Monaten für die klinische Prüfung angestrebt.

## Einschlusskriterien

In die Studie werden insgesamt 150 Patienten aufgenommen, die folgende Einschlusskriterien erfüllen:

1. Männliche und/ oder weibliche Studienteilnehmer im Alter zwischen 30 und 75 Jahren
2. Bereitschaft des Studienteilnehmers, den Anweisungen des Prüfarztes zu folgen, d.h. die Studienbedingungen einzuhalten
3. Vorliegen der klinischen Diagnose „chronische Schmerzen der Lendenwirbelsäule“ (LWS) (chronische lokale oder pseudoradikuläre Schmerzen im Bereich der LWS aufgrund z.B. einer degenerativen Grunderkrankung mit Ausschluss der unten genannten Diagnosen)
4. Schmerzen der LWS seit mindestens 12 Monaten (= chronische LWS-Schmerzen), die therapeutischen Standardtherapiemaßnahmen müssen ausgeschöpft sein
5. Durchschnittswert der Schmerzintensität bezogen auf die letzten 7 Tage ≥ 40 mm auf einer visuellen Analogskala von 0-100 mm
6. In den letzten 4 Wochen nur medikamentöse Therapie der LWS-Beschwerden (Bedarfsbehandlung mit peripher wirkenden Analgetika, nichtsteroidalen Antiphlogistika und Muskelrelaxantien)
7. Bereitschaft einer ausreichenden/effektiven Kontrazeption bei Frauen im gebärfähigen Alter. Es gelten kontrazeptive Maßnahmen als effektiv, wenn die Versagerate <1%/Jahr beträgt, z.B. orale hormonelle Verhütung mit Kombinationspräparaten, Implantate, Intrauterinpessar, Diaphragmapessar, Portioklappe oder sexuelle Abstinenz.
8. Schriftliche Einwilligungserklärung

## Ausschlusskriterien

In die Studie werden insgesamt 150 Patienten aufgenommen, auf die folgende Ausschlusskriterien nicht zutreffen:

1. Bereits zuvor oder aktuell durchgeführte Behandlung mit Disci-Präparaten
2. Bedarfsbehandlung der Schmerzen mit anderen als peripher wirkenden Analgetika und nichtsteroidalen Antiphlogistika (NSAR)
3. Regelmäßige (> 1/Woche) Einnahme von Schmerzmitteln aufgrund von anderen Erkrankungen
4. Protrusion oder Prolaps einer/mehrerer Bandscheibe(n) mit neurologischer Symptomatik
5. Vorausgegangene Wirbelsäulenoperationen
6. V.a. infektiöse Spondylopathie
7. LWS-Beschwerden aufgrund einer malignen und entzündlichen Erkrankung
8. Organische Ursachen der Beschwerden wie z.B. Morbus Bechterew, Morbus Reiter oder Morbus Behçet
9. Angeborene Missbildungen der Wirbelsäule mit Ausnahme leichter Lordosen, Kyphosen oder Skoliosen
10. V.a. Osteoporose mit Kompressionsfraktur eines oder mehrerer Wirbelkörper
11. V.a. spinale Stenosen
12. Spondylolyse oder Spondylolisthesis
13. Physiotherapie in den letzten 4 Wochen vor Studienbeginn oder während der Studie geplant
14. Beginn einer neuen Therapie zur Behandlung der LWS-Schmerzen in den vergangenen 4 Wochen
15. Bereits geplante neue Therapie (inklusive Operationen) zur Behandlung der LWS-Schmerzen im Studienzeitraum
16. Komplementärmedizinische Therapie (z.B. Akupunktur, Homöopathie, Phytotherapie, Neuraltherapie) in den letzten 4 Wochen vor Studienbeginn oder während der Studie geplant
17. Patienten, bei denen davon auszugehen ist, dass sie aus sprachlichen, intellektuellen bzw. anderen Gründen nicht in der Lage sind, die Bedeutung der klinischen Studie zu erkennen und die dafür notwendige Compliance aufzubringen
18. Patienten mit Alkohol-, Medikamenten- und/oder Drogenabhängigkeit
19. Gleichzeitige Teilnahme an anderen klinischen Studien. Die letzte Teilnahme an einer klinischen Studie muss mindestens 3 Monate zurückliegen
20. Vorliegen einer schwerwiegenden und/oder akuten oder chronischen organischen oder psychischen Erkrankung mit starker Beeinträchtigung des Allgemeinbefindens, die eine regelmäßige Studienteilnahme nicht möglich machen
21. Patienten mit Gerinnungsstörungen oder unter Einnahme gerinnungshemmender Medikamente
22. Schwangere Patientinnen (Vorliegen eines positiven Schwangerschaftstests bei der Einschlussuntersuchung) und Stillende
23. Patienten mit laufendem Rentenantrag
24. Studienteilnehmer, die mit der Planung und Durchführung der Studie betraut sind
25. Bekannte Überempfindlichkeit gegen einen der Inhaltsstoffe

## Erläuterung der Auswahlkriterien der Studienteilnehmer

Alle potentiell für die klinische Studie geeigneten Studienteilnehmer werden zum Nachweis, dass die Studienpopulation ohne Selektion ausgewählt wurde, vom Prüfarzt auf einem Pre-Study Screening Log dokumentiert, aus dem hervorgeht, wie viele Studienteilnehmer gescreent und aus welchen Gründen sie eventuell nicht in die Studie aufgenommen wurden.

Eingeschlossen in die Studie werden Patienten beiderlei Geschlechts im Alter von 30 – 75 Jahren mit der Diagnose chronisch unspezifische LWS-Schmerzen. Bei jüngeren Patienten ist die Prävalenz unspezifischer LWS-Schmerzen eher gering. Bei Patienten, die älter als 75 Jahre sind, ist es wahrscheinlich, dass die angewendeten Messinstrumente nicht mehr valide erhoben werden können und die Möglichkeit einer relevanten Komorbidität steigt.

Die Intensität der Schmerzen sollte mindest mittelstark bis stark sein (≥ 40 mm auf einer 100 mm VAS Schmerzintensität), da ansonsten aufgrund des gewählten Zielparameters kaum messbare Veränderungen zu erwarten sind.

Ausgeschlossen von der Studie werden insbesondere Patienten, deren LWS-Schmerzen nicht auf unspezifische Rückenschmerzen sondern auf andere organische und psychische Ursachen zurückzuführen sind. Durch eine Spezifizierung der Diagnose auf „chronisch unspezifische LWS- Schmerzen“ soll ein homogenes Patientengut aufgenommen werden. LWS-Schmerzen aufgrund von anderen Erkrankungen unterscheiden sich in Symptomatik, Diagnostik bzw. Diagnose und Therapie von chronisch unspezifischen Rückenschmerzen.

Ausgeschlossen von der Studie werden weiterhin Patienten, die eine andere Therapie als NSAR einnehmen/durchführen oder durchführen werden und die bereits mit Disci/Rhus toxicodendron comp.® therapiert wurden. Bei Patienten, die andere Therapiemaßnahmen als NSAR zu sich nehmen (z.B. zentralnervös wirkende Analgetika) oder anwenden (z.B. Physiotherapie) kann aufgrund von auftretenden Misch-Therapieeffekten die Wirksamkeit der Prüftherapie nicht korrekt beurteilt werden, zudem schränkt beispielsweise die Therapie mit zentralnervös wirkenden Analgetika die Beurteilungsfähigkeit ein. Patienten, die bereits mit Disci/Rhus toxicodendron comp.® behandelt wurden, können nicht mehr adäquat verblindet werden, da sie möglicherweise das Prüfpräparat erkennen.

Explizit ausgeschlossen werden Patienten mit Erkrankungen oder mit Lebenssituationen, die die Ergebnisse der klinischen Prüfung verfälschen könnten, Patienten, für die die Teilnahme medizinisch riskant wäre und Patienten, bei denen aus ethischen Gründen eine Teilnahme nicht möglich ist.

## Erläuterung zur Geschlechterverteilung der Studienteilnehmer

Die klinische Prüfung wird durchgeführt mit Patienten beiderlei Geschlechts. Die durchgeführte Therapie wird nicht geschlechtsspezifisch angepasst. Es wird nicht erwartet, dass das Geschlecht der Studienteilnahme einen Einfluss auf das Behandlungsergebnis hat. Aufgrund nicht ausreichender embryotoxischer bzw. toxischer Daten dürfen schwangere und stillende Frauen an der Studie nicht teilnehmen. Frauen, die bereit sind, eine effektive Kontrazeption zu berücksichtigen (CPMP/ICH/286/95), werden eingeschlossen. Es gelten kontrazeptive Maßnahmen als effektiv, wenn die Versagerate <1%/Jahr beträgt, z.B. orale hormonelle Verhütung mit Kombinationspräparaten, Implantate, Intrauterinpessar, Diaphragmapessar, Portioklappe oder sexuelle Abstinenz.

## Abbruch der klinischen Studie beim Studienteilnehmer

Die Studienteilnehmer haben das Recht, die Studie jederzeit ohne Angabe von Gründen abzubrechen.

Ebenso haben der Prüfarzt, die Studienzentrale oder die WALA Heilmittel GmbH das Recht, Studienteilnehmer aus Sicherheitsgründen oder aus Gründen einer validen Datenerhebung von einer weiteren Studienteilnahme auszuschließen.

### Abbruchkriterien

In der vorliegenden Studie wird unterschieden zwischen Therapieabbruch und Studienabbruch. Bei

Therapieabbruch erhalten die Patienten weiterhin Patientenfragebögen, dies ist bei einem

Studienabbruch nicht der Fall.

Ein Therapieabbruch kann, muss aber nicht einen Studienabbruch nach sich ziehen.

Gründe für einen individuellen Therapieabbruch können sein:

- Freiwilliger Abbruch zu jedem Zeitpunkt der Therapie durch den Studienteilnehmer, ohne Angabe von Gründen und ohne dass ihm ein Nachteil bezüglich der weiteren Behandlung widerfährt
- Auftreten von unerwünschten Ereignissen, die eine sofortige Behandlung mit Medikamenten notwendig machen, die zur Beeinträchtigung der Aussagefähigkeit der Testergebnisse führen
- Begründete Gefährdung der Sicherheit des Studienteilnehmers aus der Sicht des Prüfarztes, der Studienzentrale und/oder der WALA Heilmittel GmbH
- Schwerwiegende Verletzung der Compliance eines Studienteilnehmers aus der Sicht des Prüfarztes, der Studienzentrale und/oder der WALA Heilmittel GmbH
- Fälschlicherweise eingeschlossener Studienteilnehmer (z.B. Ein- und Ausschlusskriterien treffen in relevanten Punkten nicht zu)
- Voraussehbares Nichteinhalten der Studienvisiten
- Studienteilnehmer ist nicht mehr auffindbar (lost to follow up)
- Schwangerschaft

Gründe für einen individuellen Studienabbruch können sein:

- Freiwilliger Abbruch zu jedem Zeitpunkt der klinischen Studie durch den Studienteilnehmer, ohne Angabe von Gründen und ohne dass ihm ein Nachteil bezüglich der weiteren Behandlung widerfährt
- Entblindung der Therapie eines Patienten unter Verwendung der Notfallumschläge

### Vorgehen bei vorzeitigem Therapie-/Studienabbruch

Bei vorzeitigem Studienabbruch ist folgendermaßen vorzugehen:

- Der Prüfarzt informiert die verantwortliche Person der WALA Heilmittel GmbH, den Monitor oder den LKP, wenn er unsicher ist, ob bei einem Studienteilnehmer die Studie abgebrochen werden muss.
- Studienteilnehmer, die die Studie von sich aus abbrechen, sollen nach dem Grund des Abbruchs oder einem bestehenden unerwünschten Ereignis befragt werden. Wenn notwendig und bei Einverständnis des Studienteilnehmers sollte eine abschließende Untersuchung durch den Prüfarzt stattfinden. Schwerwiegende unerwünschte Ereignisse müssen nachverfolgt werden.
- Die Nachbeobachtung oder Behandlung soll in angemessener Weise gesichert werden.
- Der Abbruch muss in der Patientenakte und im entsprechenden Abschnitt des CRF mit Datum und Angabe des Grundes, der zum Studienabbruch geführt hat, dokumentiert werden. Konnte kein Abbruchsgrund ermittelt werden, muss dies dokumentiert werden.
- Der CRF eines ausgeschiedenen Teilnehmers wird von außen als Drop out gekennzeichnet und verbleibt im Prüfzentrum.
- Vorzeitig ausgeschiedene Studienteilnehmer werden nicht ersetzt.
- Wenn der Studienteilnehmer einverstanden ist, sollen alle notwendigen Untersuchungen gemäß Prüfplan durchgeführt und alle unerwünschten Ereignisse und jegliche Begleitmedikation dokumentiert werden. Es ist darauf zu achten, das Prüfpräparat einzusammeln und das Drug Accountability Formblatt zu vervollständigen.

### Verfahren nach dem individuellen Studienende

Falls nach dem individuellen Studienende eine Weiterbehandlung erforderlich sein sollte, richtet sich diese nach dem medizinischen Befund und liegt in der Verantwortung des Prüfarztes, der in der Behandlung und Überwachung von Patienten mit chronischen LWS-Schmerzen geschult ist.

Die Studienteilnehmer, die die Studie bzw. Therapie abbrechen, werden darauf hingewiesen, dass sie sich bis 30 Tage nach dem individuellen Studienende bei Reaktionen im Testareal wie auch beim Auftreten von unerwünschten Ereignissen mit dem Prüfarzt in Verbindung setzen sollen.

# Prüfpräparate

## Prüfpräparate

### Beschreibung und Zusammensetzung der Prüfpräparate

#### Disci/Rhus toxicodendron comp.® (Verum und Warteliste)

| Darreichungsform | Flüssige Verdünnung zur Injektion | |
| --- | --- | --- |
| Art der Anwendung | zur subcutanen Injektion | |
| Hersteller | WALA Heilmittel GmbH | |
| Wirkstoffe | Aconitum napellus e tubere ferm 33c Dil. D4  Argentum metallicum Dil. D18 aquos.  Arnica montana e planta tota ferm 33c Dil. D18  Disci intervertebrales bovis (cervicales, thoracici et lumbales) Gl Dil. D6  Formica rufa ex animale toto Gl Dil. D5  Gelsemium sempervirens e rhizoma ferm 35b Dil. D2  Granit Dil. D8  Leontopodium alpinum e planta tota ferm 36 Dil. D2  Mandragora officinarum e radice ferm 34d Dil. D4  Phyllostachys e nodo ferm 35c Dil. D4  Toxicodendron quercifolium e foliis ferm 33d Dil. D4 | 0,1 g  0,1 g  0,1 g  0,1 g  0,1 g  0,1 g  0,1 g  0,1 g  0,1 g  0,1 g  0,1 g |
| Sonstige Bestandteile | Natriumchlorid, Natriumhydrogencarbonat, Wasser für Injektionszwecke | |

Bei dem Prüfpräparat für die Verum- und Wartelistengruppe handelt es sich um ein zugelassenes Arzneimittel, das für die klinische Prüfung abweichend von dem im Handel befindlichen Fertigarzneimittel gemäß den Erfordernissen der klinischen Prüfung gekennzeichnet ist. (**siehe 5.1.5)**

#### Isotone Lösung (Placebo)

| Darreichungsform | Flüssige Verdünnung zur Injektion |
| --- | --- |
| Art der Anwendung | zur subcutanen Injektion |
| Hersteller | WALA Heilmittel GmbH |
| Wirkstoffe | keine |
| Sonstige Bestandteile | Natriumchlorid, Natriumhydrogencarbonat, Wasser für Injektionszwecke |

Bei dem Prüfpräparat für die Placebogruppe handelt es sich um eine isotone Lösung, die gemäß den Erfordernissen der klinischen Prüfung gekennzeichnet ist.

### Art der Anwendung

Pro Behandlung werden 10 ml (1 Ampulle) appliziert. Die Applikation erfolgt s.c. unter der Verwendung von 0,4 mm Nadeln und 10 ml Spritzen in 5-10 Teilmengen an mehreren Stellen im Schmerzbereich der LWS.

Verumgruppe:

Die Patienten der Verumgruppe erhalten 12 Veruminjektionen: 8 in den ersten 4 Wochen (2x/Woche im Abstand von mind. einem Tag ohne Therapie) und 4 in den letzten 4 Wochen (1x/Woche im Abstand von mind. drei Tagen ohne Therapie). Injiziert wird pro Behandlung eine 10 ml Ampulle in 5 bis 10 Teilmengen.

Placebogruppe:

Die Patienten der Placebogruppe erhalten 12 Placeboinjektionen: 8 in den ersten 4 Wochen (2x/Woche im Abstand von mind. einem Tag ohne Therapie) und 4 in den letzten 4 Wochen (1x/Woche im Abstand von mind. drei Tagen ohne Therapie). Injiziert wird pro Behandlung eine 10 ml Ampulle in 5 bis 10 Teilmengen.

Wartelistengruppe:

Den Patienten der Wartelistengruppe wird nach Abschluss der Wartezeit eine Behandlung mit Disci/Rhus toxicodendron comp.® angeboten. Das angebotene Dosierungsschema entspricht dem der Verumgruppe, wird jedoch nicht mehr im Rahmen der Studie evaluiert.

### Herstellung

Die Prüfpräparate werden gemäß den Regeln der guten Herstellungspraxis (Good Manufacturing Practice – GMP) hergestellt, geprüft und freigegeben.

### Kennzeichnung der Prüfpräparate

Die Studienmedikation wird pro Patient mit einer zufällig generierten dreistelligen Nummer versehen, die der Randomisierungsnummer entspricht.

Weiterhin sind folgende Informationen auf der Studienmedikation enthalten:

10 ml Ampulle

DISCI-Studie/DISRHU-1

10 ml, Zur subc. Injektion

Zur klinischen Prüfung bestimmt!

Ch.B.   "6-stellig"

Verw. bis:

WALA

Exemplarisch 01-567

Faltschachtel

DISCI-Studie/DISRHU-1

5 Ampullen mit je 10 ml Flüssige Verdünnung zur Injektion

Zur klinischen Prüfung bestimmt

Art der Anwendung:

Zur subcutanen Injektion

Dosierung:

4 Wochen lang 2-mal wöchentlich und anschließend 4 Wochen lang 1-mal wöchentlich 1-mal täglich eine Ampulle zu 10 ml flüssiger Verdünnung zur Injektion in 5-10 Teilmengen an mehreren Stellen im Schmerzbereich der LWS injizieren. Bitte Prüfplan beachten!

EudraCT-Nr.: 2006-006390-24

Ch.-B.:

Verwendbar bis:

Gebrauchte und nicht verwendete Prüfpräparate nach Beendigung der Studie bitte dem Hersteller zurückgeben! Prüfpräparate nach Ablauf des auf dem Behältnis angegebenen Verfallsdatum nicht mehr anwenden.

Sponsor:

WALA Heilmittel GmbH

Dorfstrasse 1

Bad Boll/Eckwälden

Telefon: 07164/930-4444, Fax: -6989

Exemplarisch 01-567

### Verpackung und Bezug der Prüfpräparate

Im Rahmen der klinischen Prüfung werden für jeden Patienten 12 Ampullen Prüfmedikation benötigt. Für jeden Patienten werden 3 Faltschachteln à 5 Ampullen, also insgesamt 15 Ampullen Prüfmedikation bereitgestellt. Die drei überzähligen Ampullen dienen als Ersatz im Falle einer Beschädigung. Jede dieser Faltschachteln ist mit der Randomisierungsnummer gekennzeichnet. Die 3 Faltschachteln für einen Patienten werden gebündelt.

Nach Initiierung eines Prüfzentrums erhält dieses automatisch eine Erstlieferung von Prüfmedikation für den Bedarf von 10 Patienten.

Rekrutiert ein Prüfzentrum mehr als diese zehn Patienten, kann der Prüfarzt nach Bedarf Prüfmedikation für je weitere 10 Patienten schriftlich bei der Firma WALA anfordern. Ein entsprechendes Bestellformular wird zur Verfügung gestellt.

### Lagerung

Die Prüfpräparate sind bei Raumtemperatur, zur Vermeidung von Verwechslungen getrennt von anderen Arzneimitteln und für Unbefugte unzugänglich aufzubewahren. Dies gilt auch für eventuelle Restmengen. Die Aufbewahrung im Lieferkarton wird empfohlen, sofern die örtlichen Gegebenheiten dies zulassen. Ein Temperature Log ist nicht notwendig.

Die Eignung des Lagerorts prüft der Monitor im Rahmen der Initiierung des Prüfzentrums.

### Umgang mit den Prüfpräparaten

Der Prüfarzt ist für die sichere und korrekte Anwendung der Prüfpräparate verantwortlich. Die Prüfpräparate sind ausschließlich für die klinische Studie zu verwenden. Sollten Veränderungen an den Prüfpräparaten festgestellt werden, ist die WALA Heilmittel GmbH umgehend zu benachrichtigen.

### Bilanzierung (Drug Accountability)

Vom Prüfarzt oder von der dafür autorisierten Person sind der Erhalt des Prüfpräparates und die Rückgabe an den Hersteller zu dokumentieren.

Der Prüfarzt oder die dafür autorisierte Person muss im Drug Accountability Log dokumentieren, welche Prüfpräparate ausgegeben wurden. Jedes vorsätzlich oder versehentlich vernichtete Prüfpräparat muss erfasst werden und Diskrepanzen zwischen Ausgabe und Rücknahme müssen nachvollziehbar sein und begründet werden.

Die angebrochenen, nicht verbrauchten sowie leeren Packungen des Prüfpräparates werden nach Überprüfung der Bilanz während des Monitorings vom Monitor versandfertig verpackt und nach Information der WALA Heilmittel GmbH wird deren Abholung veranlasst. Der Hersteller ist autorisiert, die Prüfpräparate zu vernichten.

### Randomisierung

Die Randomisierung erfolgt, nachdem die Patienten aufgeklärt wurden, die Einwilligungserklärung unterschrieben und den Patientenfragebogen 1 ausgefüllt haben. Die Studienärzte erhalten Briefumschläge in aufsteigender Nummerierung, die jeweils eine Randomisierungsnummer enthalten. Die Randomisierungsnummer entspricht dem Patientencode und der Nummer der Studienmedikation für den jeweiligen Patienten.

Wenn ein Studienteilnehmer die klinische Prüfung abbricht, darf die vergebene Randomisierungsnummer nicht nochmals vergeben sowie der Studienteilnehmer nicht nochmals in die klinische Prüfung eingeschlossen werden.

### Verblindung

Die Zuordnung der Behandlung erfolgt in dieser klinischen Prüfung für den Vergleich Verum vs. Placebo doppelblind. Verblindet sind der Patient und der Prüfarzt, zudem wird der Statistiker (Auswertung der primären Zielgröße) verblindet.

Die klinische Prüfung wird doppelblind durchgeführt. Um die Verblindung sicherzustellen, werden entsprechende, vom Verumpräparat nicht zu unterscheidende, Placebopräparate vom Hersteller zur Verfügung gestellt. Die Zuordnung der Prüfmedikation erfolgt anhand der Randomisierungsnummer, die auf den Faltschachteln und Ampullen aufgebracht wird. Weder für den Prüfarzt, noch für den Patienten, den Monitor und die Mitarbeiter der Studienzentrale ist ersichtlich, um welche Behandlungsgruppe es sich handelt, mit Ausnahme der Wartelistengruppe. Zudem haben diese Personen keinen Zugang zur Randomisierungsliste, mit der Ausnahme einer Notfallsituation für den einzelnen Patienten (Notfallumschlag).

### Entblindung

Eine Entblindung ist zu jedem Zeitpunkt der klinischen Prüfung sichergestellt.

Die Entblindung erfolgt während der klinischen Prüfung nur in Notfällen, z.B. kann das Auftreten eines SUE’s eine Entblindung notwendig machen. Alle Prüfzentren erhalten versiegelte Notfallumschläge für jeden Patienten, die eine Entschlüsselung der Therapie eines Patienten durch den Prüfarzt im Notfall möglich machen.

Im Falle einer Entblindung muss der Prüfarzt diese unter Angabe einer Begründung dokumentieren. Innerhalb eines Werktages nach Entblindung faxt der Prüfarzt die Meldung zur Entblindung des Patienten an die Studienzentrale, welche von dort der Wala Heilmittel GmbH per Fax gemeldet wird. Die Meldung zur Entblindung beinhaltet Patientencode, Geburtsjahr, Datum der Entblindung, Zentrum und Prüfarzt und den Grund der Entblindung.

Die WALA Heilmittel GmbH und die Studienzentrale behalten sich das Recht vor, bei jedem SUE, bei dem ein Verdacht auf kausalen Zusammenhang mit dem Prüfpräparat besteht und welches gemäß GCP-V an die Behörden und Ethikkommissionen gemeldet werden muss, den betreffenden Patienten zu entblinden.

### Nebenwirkungen, Wechselwirkungen und Gegenanzeigen

Nebenwirkungen und Wechselwirkungen von Disci/Rhus toxicodendron comp.® sind nicht bekannt.

Folgende Gegenanzeigen sind bekannt:

Das Arzneimittel soll nicht angewendet werden bei Überempfindlichkeit gegen das Spendertiereiweiß (Eiweiß vom Spendertier Rind). Das Arzneimittel sollte bei bekannter Überempfindlichkeit gegen Giftsumachgewächse oder einen der übrigen Bestandteile nicht angewendet werden.

## Begleitbehandlung (vor-, während-, nach der klinischen Prüfung)

**Erlaubte Begleitmedikation**

Während der Studie können akute Schmerzen bei Bedarf mit nichtzentralwirksamen Analgetika (z.B. Paracetamol) und nicht-steroidalen Antiphlogistika (z.B. Ibuprofen und Diclofenac) behandelt werden. Nach Möglichkeit sollten die gleichen Medikamente wie in den letzten 4 Wochen vor Einschluss in die Studie verwendet werden. Die Patienten dokumentieren diese Bedarfsmedikation in den ersten 8 Wochen täglich in einem Patiententagebuch. Die Patienten werden hierzu vom Prüfarzt bei jeder Behandlung sowie nach 26 Wochen retrospektiv befragt.

**Nicht erlaubte Begleitmedikation und -behandlung**

Nicht erlaubt als Begleitmedikation ist die Behandlung mit Analgetika und anderen Medikamenten, die über das Zentrale Nervensystem (ZNS) wirken. Nicht erlaubt ist die Behandlung mit physikalischer Medizin, manueller Therapie und Krankengymnastik. Nicht erlaubt ist die Behandlung mit Therapieverfahren der Komplementärmedizin (wie z.B. Akupunktur, Massage, Spinale Manipulation, Neuroreflexotherapie, Phytotherapie, Chinesischer Arzneimitteltherapie, Homöopathie, Arzneimitteln der anthroposophischen Medizin mit Ausnahme des Prüfpräparates). Weiterhin sollten keine elektiven Operationen an der Wirbelsäule geplant werden.

## Compliance

Der Studienteilnehmer ist darauf hinzuweisen, dass er regelmäßig zur Behandlung erscheinen soll. Der Prüfarzt sieht anhand der Einhaltung der Behandlungstermine, wie compliant der Patient ist. Jede Unterbrechung der Behandlung ist anhand der Behandlungsdaten dokumentiert.

Die Beurteilung der Compliance der Patienten beim Ausfüllen der Patientenfragebögen bzw. der Patiententagebücher wird durch die Studienzentrale mit einer regelmäßigen Abfrage der Datenbanksoftware (Access) überprüft. Nicht rechtzeitig eingegangene Fragebögen werden in einem standardisiertem Mahnverfahren angemahnt. Eingehende Patientenfragebögen und Patiententagebücher werden unmittelbar nach Eingang auf Vollständigkeit untersucht und fehlende Angaben (Missings) werden zeitnah telefonisch nachgefragt.

Falls die Studienzentrale primär die Information vom Patienten erhält, dass dieser die Studie während der Therapiephase abbricht, wird umgehend innerhalb eines Werktages der Prüfarzt über dieses Ereignis informiert und um Dokumentation dieses Vorgangs gebeten. Auch die Studienzentrale vermerkt jeden Therapie- und Studienabbruch in der Datenbank.

# Dokumentationsinstrumente

Für jeden Studienteilnehmer ist bei Beginn der Studie ein Arzt Case Report Form (CRF) anzulegen, in dem die im Folgenden beschriebenen Informationen standardisiert dokumentiert werden. Ein komplett ausgefülltes Arzt CRF besteht aus:

- Pre-Study Screening Log
- Einschlussuntersuchung
- Faxmeldung Patienteneinschluss
- Patientenidentifikation
- Therapiedokumentationen
- Befragung nach der 3. Behandlung
- Bericht über SUE
- Bericht über UE
- Therapie-/Studienabbruch
- Entblindung
- Schwangerschaft Outcome-Bericht
- Überdosierungsbericht
- Drug Accountability

Das Patienten CRF besteht aus:

- Einwilligungserklärung
- Patientenfragebogen 1-3
- Patiententagebuch 1-3
- Befragung nach der 3. Behandlung

Der Prüfarzt wird gebeten, dass Arzt CRF (soweit notwendig) vollständig und leserlich mit schwarzem Kugelschreiber auszufüllen. Ggf. notwendige Ergänzungen und Korrekturen müssen vom Prüfarzt mit Datum und Namenskürzel abgezeichnet werden. Falsche Einträge dürfen dabei nur einmal durchgestrichen werden, so dass der alte Eintrag gut lesbar bleibt. Sie dürfen nicht mit Korrekturmitteln (z.B. Tipp-Ex) beseitigt werden. Der Prüfarzt bittet den Patienten, die Patientenfragebögen und Patiententagebücher sowie die Patientenbefragung nach der 3. Behandlung vollständig und leserlich auszufüllen.

Die ausgefüllten Patienten CRFs werden an die Studienzentrale gesandt, der Arzt erhält keine Kopien der ausgefüllten Patientenfragebögen und Patiententagebücher. Die vollständig ausgefüllten Arzt- und Patientenunterlagen werden nur in der Studienzentrale vollständig dokumentiert. Im Note to File wird im CRF Ordner darauf verwiesen, dass die ausgefüllten Patientenfragebögen und Patiententagebücher in der Studienzentrale liegen.

## Pre-Study Screening Log

Jeder interessierte Patient wird im Pre-Study Screening Log dokumentiert.

Der Pre-Study Screening Log enthält pro interessierten Patienten eine laufende Screeningnummer, aktuelles Datum, Angaben zur Person (Name, Vorname, Geburtsdatum, Geschlecht), Aufnahme in die Studie ja/nein, evtl. Grund der Nichtaufnahme.

## Einwilligungserklärung

Der Studienteilnehmer sowie der Prüfarzt müssen die Einwilligungserklärung vor Beginn der studienspezifischen Untersuchungen eigenhändig datieren und unterschreiben.

## Patientenidentifikation

Die Patientenidentifikation ermöglicht dem Prüfarzt und dem Studienmonitor die schnelle Identifizierung eines Patienten durch die Zuordnung des Patientencodes zum Namen des Patienten. Auf der Patientenidentifikation werden folgende Angaben zu allen Patienten dokumentiert: Name, Vorname und Geburtsdatum des Patienten, Patientencode = Randomisierungsnummer bzw. Warteliste.

Dabei wird pro Patient ein Formular verwendet.

## Faxmeldung „Patienteneinschluss“

Dieses Formular wird innerhalb von 24 Stunden nach Einschluss eines neuen Patienten vom Prüfarzt an die Studienzentrale gefaxt. Es enthält Einschlussdatum, Patientencode und Name des Prüfarztes sowie Interventions- oder Wartelistengruppe.

## Schmerzskala (VAS)

Die Wirksamkeit von Disci/Rhus toxicodendron comp.® vs. Placebo bzw. vs. Wartelistengruppe erfolgt anhand einer visuellen Analogskala ((VAS) 0-100 mm)^5^, auf der die durchschnittlichen Schmerzen bezogen auf die letzten 7 Tage erfasst werden. Die Angabe erfolgt über die Patienten zu Baseline, nach 8 (primäre Zielgröße) sowie nach 26 Wochen (sekundäre Zielgröße).

Die VAS ist das Instrument zur Messung von subjektiven Schmerzzuständen^5^. Die subjektiv empfundene Schmerzintensität gibt der Patient auf einer unskalierten Strecke zwischen zwei Punkten an. Der Abstand beträgt 10 cm = 100 mm. 0 mm bedeutet, dass der Patient keine Schmerzen hat, 100 mm bedeutet, dass der Patient maximal vorstellbare Schmerzen hat.

Der Patient legt auf dieser Strecke durch ein Kreuz die Schmerzintensität fest.

X

0 100

kein Schmerz maximal vorstellbarer Schmerz

## Einschlussuntersuchung

Der Bogen Einschlussuntersuchung enthält Fragen zur Person, zu Anamnese, klinischem Befund, Diagnose genauer spezifiziert (bzgl. Lokalisation und evtl. Ausstrahlung) und ggf. Begleiterkrankungen und deren Behandlung, Ein- und Ausschlusskriterien und die Einteilung des Chronifizierungsgrades mithilfe eines Standardbogens.^21^ Weiterhin wird die Erwartungshaltung des Arztes bezogen auf den Therapieerfolg des jeweiligen Patienten erhoben.

## Therapiedokumentationen

Bei jeder Behandlung werden die Anzahl der injizierten Teilmengen, die Lokalisation der Injektionen, die Behandlungsdauer und das Vorhandensein unerwünschter Ereignisse (UE) und/oder das Auftreten eines schweren unerwünschten Ereignisses (SUE) sowie ggf. Änderung der Begleittherapie bzw. -medikation im Formblatt Therapiedokumentation dokumentiert.

In der 12. Therapiedokumentation wird die Einschätzung zur Wirksamkeit der Behandlung vom Arzt angegeben.

## Befragung nach der 3. Behandlung

Nach der dritten Behandlung gibt der Arzt dem Patienten das Formular „Patientenbefragung nach der 3. Behandlung“ aus (nach Vincent 1990^22^), das primär dazu dient, durch vier Fragen die Glaubwürdigkeit der Interventionen und damit indirekt die Verblindung zu beurteilen. Weiterhin beantwortet der Arzt ein separates Formular mit diesen Fragen.

## Patiententagebuch

In drei Tagebüchern (Patiententagebuch 1 – Woche 1-2, Patiententagebuch 2 – Woche 3-4, Patiententagebuch 3 – Woche 5-8) dokumentiert der Patient (in allen drei Gruppen) in den ersten 8 Wochen nach Randomisation, ob er am jeweiligen Tag an Rückenschmerzen litt, und wenn ja, was er gegen die Schmerzen unternommen hat (Anzahl von Tagen mit Akutmedikationsbedarf in den Wochen 1-8 und Anzahl von Arztbesuchen aufgrund von Rückenschmerzen in den Wochen 1-8). Weiterhin werden in den Tagebüchern auch die Anzahl der Arbeitsunfähigkeitstage bei erwerbstätigen Studienteilnehmern in den Wochen 1-8 dokumentiert.

Die durchschnittlichen Schmerzen bezogen auf die letzten 7 Tage (visuelle Analogskala (0-100 mm))^5^

dokumentiert der Patient nach 4 Wochen.

## Patientenfragebögen 1-3

Die Patientenfragebögen enthalten eine modifizierte Version des von der Deutschen Gesellschaft zum

Studium des Schmerzes (DGSS) entwickelten Fragebogens für Schmerzpatienten^23^.

Dieser umfasst insbesondere:

- Visuelle Analog-Skala: Beurteilung der durchschnittlichen Schmerzen^5^ bezogen auf die letzten 7 Tage nach 26 Wochen (siehe Punkt 6.5).
- Rückenfunktion (Funktionsfragebogen Hannover – Rücken FFbH-R^1^) nach 8 und 26 Wochen.

Der FFbH-R besteht aus 12 Items, die sich vorwiegend mit den Einschränkungen im Alltagsleben befassen. Ausgewertet wird der FFbH-R über die Bildung eines Summenindex. Die Skala ist so gepolt, dass ein hoher Wert eine geringe Einschränkung bedeutet.

- Emotionale Schmerzbewertung (Schmerzempfindungsskala SES^3^) nach 8 und 26 Wochen.

Die Schmerzempfindungsskala bewertet über 2 Summenskalen mit insgesamt 24 Items sensorische und affektive Aspekte der Schmerzen.

- Beurteilung der Beeinträchtigung im Alltag (Pain Disability Index^4^) nach 8 und 26 Wochen.

Zur Messung der Beeinträchtigung der Funktion durch Schmerzen wird der PDI verwendet (Pain Disability Index). Die 7 Items des Instruments beziehen sich auf die Beeinträchtigung von familiären, häuslichen Verpflichtungen, Erholung, sozialer Aktivität, Beruf, Sexualleben, Selbstversorgung und lebensnotwendigen Tätigkeiten durch die Schmerzen.

- Beurteilung der Lebensqualität mithilfe des Fragebogens SF-36^2^ nach 8 und 26 Wochen.

Zur Messung der allgemeinen gesundheitsbezogenen Lebensqualität wird der SF-36 verwendet. Bei dem SF-36 handelt es sich um einen 36 Items umfassenden Fragebogen, der über die Bildung von 8 Subskalen (Psychisches Wohlbefinden, Körperliche Funktionsfähigkeit, Körperliche Rollenfunktion, Körperliche Schmerzen, Allgemeine Gesundheitswahrnehmung, Vitalität, Soziale Funktionsfähigkeit, Emotionale Rollenfunktion) sowie einer körperlichen und einer psychischen Summenskala ausgewertet wird. Es wurde die Version verwendet, welche sich auf die vergangene Woche bezieht.

- Erwartungshaltung des Patienten: In zwei Fragen wird die Erwartungshaltung des Patienten, bezogen auf die Behandlung mit Disci/Rhus toxicodendron comp.®, erhoben.

Zusätzlich werden im ersten Fragebogen soziodemographische Angaben, Fragen zur Erwartungshaltung des Patienten und in allen drei Fragebögen Angaben zur medizinischen Begleitbehandlung und

-medikation erhoben.

## Entblindung

Das Entblindungsformular enthält Angaben zur Gruppenzuordnung. Der Prüfarzt dokumentiert den Grund der Entblindung sowie den eventuellen Kausalzusammenhang zur Therapie

## Therapie- und Studienabbruch

Auf dem Formular Therapie- und Studienabbruch ist zu dokumentieren, ob es sich um einen Therapie- oder Studienabbruch handelt und was die Ursachen für den Abbruch sind.

## Bericht über UE

Dieses Formular umfasst die Dokumentation aller eventuell auftretenden UEs während des Studienverlaufes bei einem Patienten. Darin wird vom Prüfarzt das Symptom bzw. die Diagnose für das jeweilige UE, sowie Beginn und Verlauf des UEs dokumentiert.

## Bericht über SUE

Aufgetretene SUEs werden anhand dieses Formulars erfasst. Hier dokumentiert der Prüfarzt sowohl die Schwere des SUEs als auch Dauer, Kausalzusammenhang zur Studientherapie, getroffene Maßnahmen und Ausgang des Ereignisses.

# Studienablauf

## Zeitplan

| **Voraussichtlicher/ Geplanter zeitlicher Rahmen der Studie** | **Datum** |
| --- | --- |
| Vorbereitungsphase (4 Monate)  (Erstellung der kompletten Studienunterlagen, Rekrutierung der Prüfzentren, Einholung der Ethikvoten, Prüfarztschulung, Genehmigung BfArM) | bis 28.02.2007 |
| Patientenrekrutierungsphase (26 Wochen) | 1.03.2007 – 31.08.2007 |
| Voraussichtlicher Zeitpunkt der Aufnahme des ersten Patienten | 1.03.2007 |
| Behandlungsphase (8 Wochen je Patient) | 1.03.2007 – 31.10.2007 |
| Follow-up Phase (18 Wochen pro Patient) | 1.05.2007 – 29.02.2008 |
| Voraussichtlicher Abschlusszeitpunkt des letzten Patienten | 29.02.2008 |
| Clean File | 1.03.2008 – 30.04.2008 |
| Auswertungsphase  (Auswertung, Erstellung des Berichts) | 1.05.2008 – 30.06.2008 |
| Studienende | 30.06.2008 |

## Studieneinschluss und Erhebung der Baselinedaten

### Vergabe der Pre-Study Screeningnummer

Allen Patienten, die zur Erstuntersuchung erscheinen, teilt der Prüfarzt auf dem Pre-Study Screening Log eine Pre-Study Screeningnummer zu und dokumentiert im Falle der Nichtaufnahme in die Studie den Grund. Die entsprechende laufende Screeningnummer wird auch auf der Einschlussuntersuchung des Patienten vermerkt. Wird der Patient in die Studie aufgenommen und randomisiert, erhält er den entsprechenden Patientencode, damit wird die entsprechende laufende Screeningnummer auf allen folgenden Unterlagen nicht mehr verwendet.

### Aufklärung und Einholung der Einwilligung

Wenn ein Patient an einer Studienteilnahme interessiert ist, wird er vom Prüfarzt ausführlich mündlich über Art, Ziel, Bedeutung, Umfang der Studie, einschließlich möglicher Risiken sowie der Freiwilligkeit der Studienteilnahme und aller Rechte und Pflichten informiert. Zusätzlich erhält er die schriftliche Patientenaufklärung mit allen Informationen zur Studie. Für die Erörterung von Fragen mit dem Prüfarzt und für die Entscheidungsfindung wird ihm ausreichend Zeit zur Verfügung gestellt.

Im Falle einer positiven Entscheidung für eine Studienteilnahme und spätestens vor der Einschlussuntersuchung unterschreibt der Patient persönlich unter Angabe des Datums die Einwilligungserklärung zur Studienteilnahme. Ebenso unterschreibt der aufklärende Prüfarzt eigenhändig unter Angabe des Datums die Einwilligungserklärung.

Die unterschriebene Einwilligungserklärung wird im Original an das Institut für Sozialmedizin, Charité – Universitätsmedizin Berlin geschickt, ein Durchschlag vom Original verbleibt im Prüfarztordner am Prüfzentrum, einen weiteren Durchschlag vom Original erhält der Studienteilnehmer.

### Erhebung der Anamnese und Einschlussuntersuchungen

Liegt die Einwilligungserklärung des Patienten unterschrieben vor, bittet der Arzt den Patienten, die Visuelle Analogskala (VAS) zu den durchschnittlichen Schmerzen auszufüllen. Ist die Markierung auf der VAS ≥ 40 mm, führt der Arzt die Anamnese und Einschlussuntersuchung durch.

Dabei wird bei Frauen im gebärfähigen Alter ein Schwangerschaftstest (24 Hilary direkt) durchgeführt, es werden Fragen zur Anamnese gestellt, die Diagnose „chronische LWS-Schmerzen“ anhand vorliegender Daten und Befunde überprüft und spezifiziert (bzgl. Lokalisation und evtl. Ausstrahlung) und vorhandene Begleiterkrankungen und deren Behandlung erfragt und im Formular Einschlussuntersuchung dokumentiert. Im Anschluss erfolgt die Einteilung des Chronifizierungsgrades mithilfe der Mainzer Stadieneinteilung.^21^

### Überprüfung der Ein- und Ausschlusskriterien

Nach Auswertung der anamnestisch (inklusive soziodemographische Daten) und der durch die körperliche Untersuchung erhobenen Daten, des Schwangerschaftstests bei Frauen im gebärfähigen Alter sowie anhand der im Rahmen der Routinediagnostik vorliegenden Daten und Befunde (Anamnese, ggf. vorliegende Röntgenuntersuchung etc.) überprüft der Prüfarzt die Ein- und Ausschlusskriterien (Abschnitt 4.2, 4.3). Patienten, bei denen nicht alle Einschlusskriterien erfüllt sind oder mindestens ein Ausschlusskriterium vorliegt, können nicht an der Studie teilnehmen.

### Patientenfragebogen 1

Der Studienteilnehmer wird gebeten, den Patientenfragebogen 1 vor der Randomisierung ohne Beisein des Arztes (z.B. im Wartezimmer) auszufüllen und dem Arzt in einem verschlossenen Umschlag auszuhändigen. Der Arzt leitet den Patientenfragebogen 1 an die Studienzentrale weiter.

### Randomisierung und Vergabe des Patientencodes

Jedes Prüfzentrum erhält die Umschläge für die Randomisierung in aufsteigender Nummerierung mit der Prüfmedikation. Der Prüfarzt teilt einen Patienten randomisiert einer Behandlungsgruppe zu, indem er den verschlossenen Randomisierungsumschlag mit der niedrigsten Nummer öffnet. Die Randomisierungnummer, die im Randomisierungsumschlag enthalten ist, ist gleichzeitig der Patientencode für die Pseudonymisierung des Patienten und die Nummer des Prüfpräparates.

Die Aufklärung des Patienten über das Ergebnis der Randomisierung erfolgt standardisiert mit folgenden Aussagen:

- - Verum- oder Placebogruppe: Sie erhalten ab sofort eine Injektionsbehandlung über 8 Wochen, ob sie in der Arznei- oder Placebogruppe sind, ist auch mir nicht bekannt.
  - Wartelistengruppe: Sie müssen zwar 6 Monate auf die Behandlung warten, aber dafür haben Sie den Vorteil, dass Sie mit 100% Sicherheit die Behandlung mit Disci/Rhus toxicodendron comp.® erhalten.

Nach der Randomisierung füllt der Prüfarzt das Formular Patientenidentifikation aus und schickt den Durchschlag zusammen mit der Einwilligungserklärung postalisch innerhalb eines Werktages an die Studienzentrale. Das Original wird im Patienten-CRF abgelegt. Ein Verweis auf die Ablage befindet sich im Prüfarztordner. Das Formular Patientenidentifikation enthält Angaben zur Person des Patienten und das Ergebnis der Randomisierung (Patientencode/bzw. Randomisierungsnummer). Weiterhin faxt der Prüfarzt innerhalb eines Werktages das Formular „Faxmeldung Patienteneinschluss“ an die Studienzentrale. Diese beinhaltet Patientencode, Datum der Einwilligungserklärung, Zentrum, Prüfarzt, Angabe ob es ein Wartelistengruppepatient ist oder nicht.

### Patiententagebuch 1

Nach der Randomisation gibt der Prüfarzt dem Patienten das Patiententagebuch 1 aus und unterweist ihn in dessen Gebrauch. Das Patiententagebuch 1 ist vom Patienten vom Ausgabetag an über 2 Wochen täglich zu führen (Aufzeichnung für die Wochen 1 bis 2 nach Randomisierung). Darin wird u.a. dokumentiert, ob der Patient am jeweiligen Tag an Rückenschmerzen litt, und wenn ja, was er gegen die Schmerzen unternommen hat.

Die Patienten aller drei Gruppen werden angewiesen, das Patiententagebuch 1 nach zwei Wochen der Studienzentrale zuzusenden.

## Behandlungsphase (Woche 1-8) in Verum- und Placebogruppe

### Therapiedokumentation

Für Patienten beider Gruppen beginnt die Behandlung, wenn möglich, am Einschlusstag. Falls dies nicht möglich ist, ist mit der Behandlung in Woche 1 nach Einschluss zu beginnen. In Woche 1, 2, 3 und 4 werden je 2 Behandlungen (im Abstand von mind. einem Tag ohne Therapie) durchgeführt, in Woche 5, 6, 7 und 8 wird je eine Behandlung (im Abstand von mind. drei Tagen ohne Therapie) durchgeführt. Vor jeder Behandlung wird eine Kurzanamnese durchgeführt. Bei jeder Behandlung werden die Anzahl der Injektionen, die Lokalisation der Injektionen, die Behandlungsdauer, die unerwünschten Ereignisse (Formblatt „Bericht über UE“) und die Veränderung der Begleittherapie bzw. -medikation im Formular Therapiedokumentation vom Prüfarzt dokumentiert. Bei der letzten Behandlung dokumentiert der Prüfarzt zusätzlich seine Einschätzung bezüglich der Verblindung. Vor der letzten Behandlung führt der Prüfarzt abschließend eine orientierende körperliche Untersuchung durch.

Der Prüfarzt oder die dafür autorisierte Person muss bei jeder Behandlung im Drug Accountability Log dokumentieren, welches Prüfpräparat eingesetzt wurde.

Tritt innerhalb der Studie bei einer Patientin eine Schwangerschaft auf, muss das Formular “Schwangerschaft Outcome-Bericht“ ausgefüllt und die Therapie abgebrochen werden.

Wird eine Entblindung notwendig, ist der Notfallumschlag zu öffnen und das Formular „Entblindung“ auszufüllen.

Im Falle einer Überdosierung wird der „Überdosierungsbericht“ ausgefüllt.

### Befragung nach der 3. Behandlung

Nach der 3. Behandlung füllen sowohl Patient als auch Arzt je ein Formular „Befragung nach der 3. Behandlung“ aus.

### Patiententagebücher 2 und 3

Der Patient erhält nach zwei Wochen von der Studienzentrale das Patiententagebuch 2 (Aufzeichnung für die Wochen 3 und 4 nach Randomisierung) inklusive der VAS, die nach der 4. Woche auszufüllen ist und nach vier Wochen das Patiententagebuch 3 (Aufzeichnung für die Wochen 5 bis 8 nach Randomisierung). Die Patienten werden angewiesen, die Tagebücher 2 und 3 nach vier bzw. acht Wochen der Studienzentrale zurückzusenden.

### Patientenfragebogen 2

Dem Patienten wird in der Woche 8 von der Studienzentrale der Patientenfragebogen 2, der u.a. auch die primäre Zielgröße (VAS zu LWS-Schmerzen) enthält, zugesandt mit der Bitte, diesen unmittelbar nach der letzten Behandlung selbstständig auszufüllen und nach Ausfüllen in einem vorfrankierten Umschlag an die Studienzentrale zurückzusenden.

## Wartephase (Woche 1-8) in Wartelistengruppe

Mit Ausgabe des Patiententagebuchs 1 beginnt für die Patienten der Wartelistengruppe die Wartezeit von insgesamt 26 Wochen.

### Patiententagebücher 2 und 3

Der Patient erhält nach zwei Wochen von der Studienzentrale das Patiententagebuch 2 (Aufzeichnung für die Wochen 3 und 4 nach Randomisierung) zuzüglich der VAS, die nach der 4. Woche auszufüllen ist und nach vier Wochen das Patiententagebuch 3 (Aufzeichnung für die Wochen 5 bis 8 nach Randomisierung). Die Patienten werden angewiesen, die Tagebücher 2 und 3 nach vier bzw. acht Wochen der Studienzentrale zurückzusenden.

### Patientenfragebogen 2

Dem Patienten wird in der Woche 8 von der Studienzentrale der Patientenfragebogen 2, der auch die primäre Zielgröße (VAS zu LWS-Schmerzen) enthält, zugesandt mit der Bitte, diesen selbstständig auszufüllen und nach Ausfüllen in einem vorfrankierten Umschlag an die Studienzentrale zurückzusenden.

## Follow up (nach 26 Wochen)

### Patientenfragebogen 3

26 Wochen nach Randomisation wird allen Patienten der Patientenfragebogen 3 zugesendet. Beigefügt ist ein vorfrankierter und adressierter Umschlag, in dem der ausgefüllte Fragebogen vom Patienten direkt an die Studienzentrale gesandt werden soll.

### Behandlungsphase für die Wartelistengruppe (Woche 26 - 34)

Die Patienten der Wartelistengruppe erhalten, wenn sie es wünschen, nach 26 Wochen die Behandlung mit Disci/Rhus toxicodendron comp.®. Diese Behandlung ist nicht mehr Teil der Studienevaluation.

# Sicherheitsparameter und Variablen

Aus Gründen der Sicherheit der Studienteilnehmer werden ab dem Studieneinschluss unerwünschte Ereignisse registriert und dokumentiert, wobei diese spontan oder auf offene Befragung durch den Prüfarzt vom Studienteilnehmer berichtet sein können.

## Definition unerwünschtes Ereignis UE (gemäß GCP-V/ ICH-GCP)

Ein UE ist jedes nachteilige Vorkommnis, das einer betroffenen Person widerfährt, der ein Prüfpräparat verabreicht wurde, und das nicht notwendigerweise in ursächlichem Zusammenhang mit dieser Behandlung steht.

Ein UE kann daher jede ungünstige und unbeabsichtigte Reaktion (einschließlich eines anomalen Laborbefundes), jedes Symptom oder jede vorübergehend mit der Applikation der Prüfpräparate einhergehende Erkrankung sein, ob diese nun mit den Prüfpräparaten in Zusammenhang stehen oder nicht.

## Definition schwerwiegendes unerwünschtes Ereignis SUE (gemäß GCP-V/ ICH-GCP)

Ein SUE ist jedes unerwünschte Ereignis, das ein oder mehrere nachfolgende Kriterien erfüllt:

- zum Tod führt
- lebensbedrohlich ist
- eine stationäre Behandlung des Studienteilnehmers oder eine Verlängerung eines stationären Aufenthaltes erforderlich macht
- zu bleibender oder schwerwiegender Behinderung oder Invalidität führt
- eine angeborene Missbildung (kongenitale Anomalie) oder einen Geburtsfehler zur Folge hat.

## Dokumentation unerwünschter Ereignisse (UE) und schwerwiegender unerwünschter Ereignisse (SUE)

UE und SUE sind für alle eingeschlossenen Patienten bis zum individuellen Studienende (Ende der Follow-up Phase Woche 26) zu dokumentieren.

Für jedes UE ist auf dem UE Formblatt Art, Zeitpunkt des Auftretens, Häufigkeit (einmalig, anhaltend, intermittierend, bei jeder Dosisgabe), die Intensität, ob schwerwiegend oder nicht (ja/nein), welche Maßnahmen bzgl. der Prüfpräparate erfolgten (keine, Dosis erhöht, Dosis vermindert, Prüfpräparat abgesetzt), Kausalitätsbeurteilung und der Ausgang (wiederhergestellt, wiederhergestellt mit Folgen, nicht wiederhergestellt, Tod, unbekannt) des Ereignisses zu dokumentieren.

Jedes SUE eines Studienteilnehmers ist auf dem SUE Formblatt vollständig zu dokumentieren.

Wenn die Meldung nicht vollständig erfolgen kann, sind die zu Beginn wichtigsten meldepflichtigen Informationen folgende:

- Benennbares unerwünschtes Ereignis (Beschreibung Ereignis)
- Identifizierbarer Prüfungsteilnehmer (Alter oder Geburtsdatum, Geschlecht, Code)
- Identifizierbare Meldequelle (Zentrum, Adresse Prüfarzt)
- Zeitlicher Zusammenhang mit der Studienteilnahme (Beginn/ Ende SUE)

Ein Follow-up Bericht ist so schnell wie möglich zu erstellen. Im Falle des Todes oder bei Lebensbedrohlichkeit ist ein Follow-up Bericht innerhalb weiterer 5 Tage zu erstellen.

**Intensität**

Die Intensität eines UE/SUE wird folgendermaßen eingestuft:

- **Leicht**  Das Symptom wird kaum vom Studienteilnehmer wahrgenommen. Es beeinflusst nicht

seine Leistungsfähigkeit und Tätigkeit. Gegen die Symptome ist eine Verschreibung von Medikamenten nicht notwendig, die Persönlichkeitsstruktur des Patienten kann dies aber notwendig machen.

- **Mäßig** Symptome von ernstzunehmender Stärke, die den Studienteilnehmer beeinträchtigen;

Ausführung täglicher Verrichtungen wird beeinflusst; eine Behandlung der Symptome kann notwendig sein.

- **Schwer** Die Symptome bewirken eine schwere Beeinträchtigung, eine Behandlung der Symptome kann notwendig sein und/oder der Studienteilnehmer wird ins Krankenhaus verlegt.

**Kausalität**

Auf dem Bericht über UE/SUE ist die Frage: „Gibt es Ihrer Einschätzung nach ausreichend Hinweise auf einen Kausalzusammenhang zwischen dem unerwünschten Ereignis und den Prüfpräparaten?“ vom Prüfarzt zu beantworten.

Für die Beurteilung des Zusammenhangs zwischen der Anwendung des Prüfpräparates und dem UE/SUE werden folgende Definitionen verwendet:

- **Sicher**
  - Ereignis oder Labortestanomalität, das einem plausiblen zeitlichen Ablauf nach Anwendung der verdächtigen Testsubstanz folgt
  - Reaktion folgt einem bekannten oder erwarteten Antwortmuster auf die verdächtige Testsubstanz und ist begründbar
  - Reaktion ist nicht erklärbar durch eine bestehende Grunderkrankung oder die Begleitmedikation
  - Plausible Absetzreaktion
  - Re-Exposition ist positiv
- **Wahrscheinlich**
  - Ereignis oder Labortestanomalität, das einem plausiblen zeitlichen Ablauf nach Anwendung der verdächtigen Testsubstanz folgt
  - Reaktion folgt einem bekannten oder erwarteten Antwortmuster auf die verdächtige Testsubstanz und ist begründbar
  - Es ist unwahrscheinlich, dass die Reaktion durch eine bestehende Grunderkrankung oder die Begleitmedikation erklärt werden kann.
- **Möglich**
  - Ereignis oder Labortestanomalität, das einem plausiblen zeitlichen Ablauf nach Anwendung der verdächtigen Testsubstanz folgt
  - Reaktion folgt einem bekannten oder erwarteten Antwortmuster auf die verdächtige Testsubstanz und ist begründbar
  - Reaktion kann leicht durch eine bestehende Grunderkrankung oder die Begleitmedikation erklärt werden
- **Nicht beurteilbar**
  - Der Bericht weist auf eine mögliche Nebenwirkung hin, die nicht bewertet werden kann, weil die Information unvollständig ist und die Daten nicht vervollständigt oder verifiziert werden können.
- **Keinen Bezug**
  - Kein kausaler Zusammenhang, es handelt sich nicht um eine Nebenwirkung

## Meldung eines SUE durch den Prüfarzt

Alle schwerwiegenden unerwünschten Ereignisse müssen vom Prüfarzt innerhalb von 24h nach Bekannt werden an die WALA Heilmittel GmbH gemeldet werden. Die Meldung sollte per Fax anhand des SUE Formblattes an den Stufenplanbeauftragten (Fax.: 07164/ 930-6989) erfolgen. Die WALA Heilmittel GmbH informiert dann umgehend die Studienzentrale.

## Meldung schwerwiegender unerwarteter Nebenwirkungen (SUSAR- suspected unexpected serious adverse reaction) durch die WALA Heilmittel GmbH

Das Auftreten schwerwiegender unerwarteter Nebenwirkungen, erwarteter schwerwiegender Nebenwirkungen mit unerwartetem Ausgang, klinisch relevante häufig auftretende erwartete schwerwiegende Nebenwirkungen sowie Ereignisse im Zusammenhang mit der Studiendurchführung und Sicherheit der betroffenen Personen meldet die WALA Heilmittel GmbH innerhalb der Meldefristen gemäß §13 GCP-Verordnung dem BfArM, der zuständigen Ethikkommission, dem Leiter der klinischen Prüfung sowie an alle Studienärzte zusammen mit der Einschätzung zur Kausalität.

Jeder Prüfarzt hat die SUSAR-Meldungen der WALA Heilmittel GmbH zur Kenntnis zu nehmen und im Prüfarztordner vollständig aufzubewahren.

## Vorzeitiger Abbruch der Studie in einem Zentrum

Folgende Gründe können zum vorzeitigen Studienabbruch in einem Prüfzentrum führen:

- Abbruch durch die Studienzentrale im Auftrag der WALA Heilmittel GmbH bei mangelnder Rekrutierung von Studienteilnehmern
- Abbruch durch die Studienzentrale im Auftrag der WALA Heilmittel GmbH bei mangelnder Compliance eines Prüfarztes
- Abbruch durch die Studienzentrale im Auftrag der WALA Heilmittel GmbH bei begründetem Zweifel an einer GCP-gemäßen Studiendurchführung

Beim vorzeitigen Studienabbruch in einem Zentrum ist folgendermaßen vorzugehen:

- Unverzügliche Information des BfArMs, der zuständigen Ethikkommission und Landesbehörde durch die WALA Heilmittel GmbH
- Unverzügliche Information aller Studienteilnehmer des Zentrums, deren Studienbeteiligung noch nicht beendet ist, durch den für das Zentrum verantwortlichen Prüfarzt
- Falls notwendig Organisation einer angemessenen Weiterbehandlung und einer angemessenen Weiterbeobachtung durch das betreffende Prüfzentrum
- Rückgabe sämtlicher Studienmaterialien vom betreffenden Prüfzentrum an die WALA Heilmittel GmbH mit Ausnahme der Studienunterlagen, die am Prüfzentrum archiviert werden

## Abbruch der gesamten klinischen Studie

Gründe für den Abbruch der gesamten Studie können sein:

- Abbruch durch die WALA Heilmittel GmbH in Absprache mit dem Leiter der klinischen Prüfung beim Auftreten von unerwünschten Ereignissen, die so schwerwiegend sind, dass das entstehende Risiko-Nutzen-Verhältnis bei einer erneuten Abwägung nicht mehr akzeptabel ist
- Abbruch durch die WALA Heilmittel GmbH in Absprache mit dem Leiter der klinischen Prüfung bei Problemen im Studiendesign
- Abbruch durch die WALA Heilmittel GmbH bei mangelnder Rekrutierung von Studienteilnehmern oder bei hoher Anzahl von Studienabbrechern wie in einer gesonderten Vereinbarung geregelt

Beim vorzeitigen Studienabbruch ist folgendermaßen vorzugehen:

- Unverzügliche Information aller Studienärzte durch die WALA Heilmittel GmbH
- Unverzügliche Information des BfArMs, der zuständigen Ethikkommission und Landesbehörden durch die WALA Heilmittel GmbH
- Unverzügliche Information aller noch teilnehmenden Studienteilnehmer durch die für ein Zentrum verantwortlichen Studienärzte
- Falls notwendig Organisation einer angemessenen Weiterbehandlung und einer angemessenen Weiterbeobachtung durch das betreffende Prüfzentrum
- Rückgabe sämtlicher Studienmaterialien von den beteiligten Studienzentren an die WALA Heilmittel GmbH mit Ausnahme der Studienunterlagen, die am Prüfzentrum archiviert werden

## Safety Board

Das Safety Board unterstützt die Gewährleistung der Sicherheit für die Studienpopulation. Die Beurteilung der SUEs wird primär vom Prüfarzt vorgenommen und von der Abteilung für Pharmakovigilanz der WALA Heilmittel GmbH überwacht, bearbeitet und klassifiziert. Zusätzlich evaluiert ein unabhängiges Safety Board alle SUE-Berichte und die Bewertung der SUSAR´s.

# Datenmanagement

Das Datenmanagement wird nach den ICH-Empfehlungen zur „Good Clinical Practice“ (GCP) und den Vorgaben des Qualitätsmanagementhandbuchs des Instituts für Sozialmedizin, Epidemiologie und Gesundheitsökonomie durchgeführt. Für die Studie werden zzgl. studieninterne Standard Operating Procedures (SOPs) erstellt.

Die Patientenverwaltung und Dateneingabe erfolgt in studienspezifischen Microsoft Access 2000 Datenbanken.

Für die Datenverwaltung wird ein Data-Managementplan erstellt, der Angaben zur Datenbankstruktur, Inhouse-Review, Data-Validation, Kodierung der Angaben und Dateneingabe enthält.

**Datenerhebung und –verarbeitung**

Alle Patientenfragebögen (Patienten-CRF) erhält der Studienteilnehmer entweder direkt vom Prüfarzt oder postalisch von der Studienzentrale. Die Arztfragebögen (Arzt-CRF) liegen den teilnehmenden Ärzten vor.

Aus Gründen des Datenschutzes sind die CRF pseudonymisiert, d.h. sie tragen den Patientencode.

Die Eintragungen in das Arzt-CRF müssen mit schwarzem Kugelschreiber vorgenommen werden und lesbar sein. Jede Korrektur im Arzt-CRF wird so ausgeführt, dass die Ersteintragung lesbar bleibt. Die korrigierte Angabe wird mit Begründung, Datum und Unterschrift versehen.

Originaldokumente für die Arzt- und Patienten-CRFs verbleiben in der Studienzentrale. Die vollständig ausgefüllten CRFs sind Eigentum der WALA Heilmittel GmbH und dürfen ohne deren schriftliche Erlaubnis nicht an Dritte weitergegeben werden. Nach Studienende verbleibt eine Kopie des Arzt-CRF im Prüfzentrum.

Die von den Studienteilnehmern erhobenen Daten werden in pseudonymisierter Form zur Datenerfassung und Auswertung an die Studienzentrale gesandt. Diese Daten werden auf Vollständigkeit und Plausibilität überprüft (Inhouse Review) und dann in die Datenbank eingegeben. Bei unplausiblen oder unklaren Daten wird der Sachverhalt mittels telefonischer oder schriftlicher Nachfrage (Query) mit dem Prüfarzt oder dem Studienteilnehmer abgeklärt und korrigiert.

Bei schriftlichen Nachfragen beim Prüfarzt wird diesem das Queryformular zugesandt. Das Original mit den vorgenommenen Änderungen/Korrekturen wird an die Studienzentrale zurück geschickt, eine Kopie verbleibt beim Prüfarzt im CRF-Ordner.

Nach erfolgter Doppelteingabe erfolgt die Erstellung des Clean File (Prüfen der Daten auf Korrektheit und Plausibilität, Abgleich der SUE-Berichte mit der Pharmakovigilanz usw.). Nach Klärung aller eventuellen Rückfragen und Unplausibilitäten wird die Datenbank geschlossen und die Daten werden für die Auswertung in das SPSS- oder SAS-Datenformat übertragen.

**Archivierung der Studienunterlagen**

Der verantwortliche Prüfarzt eines Zentrums sichert die Archivierung des Prüfarztordners, der Patientenidentifikationen, Kopien der CRF und von allen weiteren, angefallenen studienrelevanten Dokumenten am Prüfzentrum für 15 Jahre nach Studienende. Die Patientenakten werden entsprechend den gesetzlichen Regelungen des Prüfzentrums aufbewahrt.

Der Trial Master File als zentraler Ordner für sämtliche im Verlauf der Studie angefallenen Dokumente (wie z.B. das Studienprotokoll, CRFs, Berichte über SUE) wird zum Teil sowohl vom Institut für Sozialmedizin, Epidemiologie und Gesundheitsökonomie als auch von der WALA Heilmittel GmbH geführt und über 15 Jahre aufbewahrt. Die Archivierung wird dokumentiert.

# Statistische Methoden und Bestimmung der Fallzahl

## Biometrische Planung und Auswertung

Durch das Design der Studie ergeben sich in den Zielgrößen verschiedene Abhängigkeiten, die bei der statistischen Auswertung berücksichtigt werden müssen. Generell werden zum einen beim einzelnen Patienten wiederholte Messungen durchgeführt, zum zweiten werden Patienten, die aus derselben Praxis stammen, möglicherweise ähnlichere Werte aufweisen als Patienten aus verschiedenen Praxen. Die Auswertungen werden daher grundsätzlich mit repeated measurement multilevel models durchgeführt (d.h. Multilevelmodell mit Messwiederholungen wenn notwendig). Die unterste Ebene ist dabei die Wiederholung, die zweite Ebene der Patient und die oberste Ebene die Praxis. Für die unterste Ebene wird als Korrelationsstruktur ein AR(1)-Prozess unterstellt (autoregressiver Prozess der Ordnung 1). Für die zweite Ebene wird eine austauschbare (‚exchangeable’) Korrelationsstruktur angenommen. Therapiegruppe und Messzeitpunkt werden als Faktoren in die Modelle eingeführt.

Folgende Kovariaten werden für die Modellbildung, gegebenenfalls Untergruppenbildung a priori festgelegt:

Patientenebene:- der jeweilige Baselinewert der betrachteten Zielgröße (entspricht einer ANCOVA)

Praxisebene: - Zentrumsgröße (groß/klein, cutoff ist vor Datenbankschließung festzulegen)

- akademischer Status (universitär vs. nicht-universitär)

- CAM-Angebot (ja/nein)

## Biometrische Definition der Zielgrößen

Die **primäre Analyse** besteht aus zwei getrennten Zwei-Gruppen-Vergleichen der Werte der Primärzielgröße zum Messzeitpunkt 8 Wochen.

1) Vergleich zwischen Verum- und Placebo-Gruppe

2) Vergleich zwischen Verum- und Wartelistengruppe

(jeweils unter Einschluss des Baselinewertes (ANCOVA)).

Die Nullhypothese für jeden der beiden Vergleiche lautet:

„Es besteht kein Unterschied zwischen den Erwartungswerten in beiden Gruppen“.

Die Alternativhypothese lautet jeweils:

„Es besteht ein Unterschied zwischen den Erwartungswerten in beiden Gruppen“.

Beide Vergleiche werden mit dem zugehörigen zweiseitigen Wald-Test zum Niveau 2,5% durchgeführt, um ein Gesamtniveau von 5% sicherzustellen. Fehlende Werte werden grundsätzlich nicht ersetzt. In einer nachfolgenden Sensitivitätsanalyse wird eine Maximum-Likelihood basierte Imputation fehlender Werte sowie eine Worst-Case-Analyse vorgenommen, um die Belastbarkeit der Ergebnisse zu untersuchen.

Für die **sekundäre Analyse** werden zusätzlich die oben genannten Praxisfaktoren ins Modell

eingeschlossen. Ferner erfolgt ein Vergleich von Placebo- und Wartelistengruppe sowie Vergleiche

der 26-Wochen-Werte ohne weitere multiple Anpassung des Testniveaus. Die Analysen der

sekundären Endpunkte erfolgen analog.

Zusätzlich zur beschriebenen prospektiv geplanten konfirmatorischen Analyse werden explorativ

weitere Kovariaten und Modifier-Variablen in die Modelle eingeführt, um Hypothesen für weitere

Forschungsarbeiten zu gewinnen.

## Fallzahlschätzung

Wenn 50 Patienten pro Gruppe eingeschlossen werden und durch eine angenommene Drop-out-Rate von 18% nach 8 Wochen noch 41 Patienten pro Gruppe für die Auswertung zu Verfügung stehen, so lässt sich ein Unterschied von 12,5 Einheiten auf der VAS und einer Standardabweichung von 18 Einheiten (Effektgröße 0,69) mit einer Power von 80% bei einem Testniveau von 5% statistisch nachweisen.

# Studienmanagement

## Monitoring

Die Prüfzentren werden von der Studienzentrale rekrutiert. Vor Beginn der klinischen Prüfung werden mit dem Prüfzentrum folgende Punkte geklärt:

- ob genügend Zeit, ausreichend und qualifiziertes Personal sowie geeignete Einrichtung/ Ausstattung (z.B. Lagermöglichkeit für die Prüfmedikation, Studienunterlagen etc.) für die Durchführung der klinischen Prüfung vorhanden sind.
- dass die klinische Prüfung gemäß Prüfplan durchzuführen ist.
- Erfüllung der Verantwortlichkeiten, welche im Vertrag festgelegt sind.
- Beachtung der studienspezifischen Dokumentation gemäß Prüfplan und Vertrag (Originaldaten, Datenerhebungsbogen).
- dass das Training des Studienpersonals am Prüfzentrum bezüglich Studiendurchführung, Dokumentation und studienspezifischer Interventionen im Rahmen des Initiierungsbesuches stattfinden wird.
- welche studienspezifischen Dokumente zur Verfügung gestellt werden müssen (Geheimhaltungserklärung, Vertrag, Lebensläufe, Einbeziehung abhängiger Personen, Eignung des Prüfzentrums, wirtschaftliche Interessen Prüfpräparat).

Das Monitoring ist gemäß der Verfahrensanweisung Y-441-VA-MO-001 „Klinisches Monitoring“ der WALA Heilmittel GmbH, den Angaben im Prüfplan durchzuführen und gemäß Y-441-DOK-MO-001 „Berichte klinisches Monitoring“ zu dokumentieren. Das Monitoring umfasst einen Initiierungsbesuch, regelmäßige Besuche sowie einen Abschlussbesuch. Der zeitliche Abstand der regelmäßigen Besuche kann jederzeit in Abhängigkeit von der Datenqualität und Rekrutierungsrate am Prüfzentrum, nach vorheriger Rücksprache mit dem Sponsor, angepasst werden.

Das Monitoring der klinischen Prüfung wird vom Koordinator Monitor der WALA Heilmittel GmbH koordiniert und von freiberuflichen Clinical Research Associates durchgeführt, die von der WALA Heilmittel GmbH beauftragt werden. Der Monitor ist wichtiges Bindeglied für die Kommunikation zwischen Sponsor, der Studienzentrale und dem Prüfarzt:

- Der Monitor ist verpflichtet, das Prüfzentrum über studienspezifische Änderungen zu informieren und bei der Durchführung zu unterstützen.
- Der Monitor überprüft, dass die Einrichtung angemessen ist und bleibt.
- Der Monitor stellt sicher, dass der Prüfarzt den gültigen Prüfplan befolgt, die Daten korrekt im Datenerhebungsbogen dokumentiert und Empfang, Anwendung und Rückgabe des Prüfpräparates dokumentiert.
- Der Monitor muss die Richtigkeit und Vollständigkeit der Einträge im Datenerhebungsbogen, der Originaldaten und der anderen prüfbezogenen Aufzeichnungen miteinander vergleichen.
- Der Monitor muss sich davon überzeugen, dass die schriftliche Einwilligungserklärung von jedem Prüfungsteilnehmer vor dessen Teilnahme an der klinischen Prüfung eingeholt wurde.

Zusätzlich zu den Verfahrensanweisungen der WALA Heilmittel GmbH ist bei der Durchführung der regelmäßigen Monitorbesuche Folgendes zu beachten:

- Jedes Prüfzentrum ist im Abstand von 6-8 Wochen zu besuchen. Je nach Datenqualität und Rekrutierungsrate ist die Frequenz, nach Rücksprache mit dem Sponsor, anzupassen.
- Der Durchschlag des Originals der Einwilligungserklärung ist vom Monitor am Prüfzentrum auf Vorhandensein zu überprüfen. Die Einwilligungserklärung besteht aus einem gelben Original und zwei weißen Durchschlägen. Ein Durchschlag verbleibt am Prüfzentrum (im Prüfarztordner), ein Durchschlag erhält der Patient und das Original wird an die Studienzentrale gesendet. Die Studienzentrale ist für die Überprüfung der Einwilligungserklärung verantwortlich.
- Der Originaldatenabgleich ist zu 100% am Prüfzentrum durchzuführen. Eine Liste über die Mindestanforderung der Originaldatendokumentation ist im Prüfarztordner zu finden.

- Die Parameter im Datenerhebungsbogen (CRF), die Source Data sind, werden festgelegt.
- Die Patiententagebücher und Patientenfragebögen müssen vom Monitor nicht überprüft werden. Der Patient erhält vom Prüfarzt den Patientenfragebogen 1 und das Patiententagebuch 1, die weiteren Patientenfragebögen (Woche 8 und 26) und -tagebücher werden ihm von der Studienzentrale zugeschickt. Der Patient schickt die ausgefüllten Patientenfragebögen und -tagebücher in vorfrankierten Rückumschlägen an die Studienzentrale. Die Studienzentrale überprüft und archiviert die Patientenfragebögen und Patiententagebücher.
- Die Anzahl der Randomisierungsumschläge und die Vollständigkeit der Notfallumschläge sind zu überprüfen.
- Im Falle eines SUEs sind keine verblindeten Arztbriefe einzusammeln (ggf. nur auf Anforderung der Pharmakovigilanz der WALA Heilmittel GmbH).
- Die Originalseiten der Einschlussuntersuchungen sind bei jedem Monitoringbesuch einzusammeln. Die Seiten sind in vorbereiteten Rückumschlägen an die Studienzentrale zu senden.
- Die Originalseiten der Therapiedokumentationen 1-12 sind zum Abschluss der jeweiligen Behandlungsphasen vom Monitor einzusammeln und in vorbereiteten Rückumschlägen an die Studienzentrale zu senden.
- Die Patientenidentifizierungsliste wird durch einzelne Patientenidentifizierungsformulare pro Patient (bestehend aus einem Original und einem Durchschlag) ersetzt. Der Durchschlag verbleibt im Prüfzentrum und das Original wird per Post an die Studienzentrale vom Prüfzentrum gesendet und archiviert.
- Der Monitor erhält wöchentlich einen Bericht über den Rekrutierungsstatus von der Studienzentrale
- Die verbrauchte und überprüfte Prüfmedikation ist am Prüfzentrum vom Monitor versandfertig zu verpacken. Der Monitor informiert die WALA Heilmittel GmbH über den möglichen Abholtermin, die Anzahl der Kartons sowie das Gewicht der Kartons, die dann die Abholung veranlasst.
- Der Clean File wird von der Studienzentrale durchgeführt. Die Studienzentrale kontaktiert bei Rückfragen direkt die Prüfzentren.

## Audit / Inspektion

Während der klinischen Studie kann jederzeit ein Audit von der WALA Heilmittel GmbH als Teil seines Qualitätssicherungssystems oder eine Inspektion durch die zuständige Behörde durchgeführt werden. Der Zweck eines Audits oder Inspektion ist unabhängig vom Monitoring, die Durchführung der klinischen Studie sowie die Einhaltung des Studienprotokolls, der SOPs, von GCP und den geltenden gesetzlichen Bestimmungen zu beurteilen.

## Training des Studienpersonals

Der verantwortliche Prüfarzt eines Zentrums ist verpflichtet, auf dem Formular Delegation der Verantwortlichkeiten im Studienteam aufzulisten, wer an der Studiendurchführung teilnimmt und welches Studienpersonal für welche Aufgaben verantwortlich ist. Der verantwortliche Prüfarzt stellt sicher, dass das Studienteam entsprechend geschult ist und ist verpflichtet, aktuelle Informationen in Bezug auf die Studiendurchführung an das Studienteam weiterzuleiten.

## Amendments

Bei einer notwendigen Änderung des Studienprotokolls wird ein Amendment erstellt. Das Amendment oder die neue Version des Studienprotokolls müssen vom BfArM genehmigt und von der zuständigen Ethikkommission bewertet werden.

Jedes Prüfzentrum ist über die Änderungen zu informieren.

# Ethik / Bundesoberbehörde

## Ethische Durchführung der klinischen Prüfung

Die klinische Prüfung wird gemäß den ethischen Prinzipien, die Ihren Ursprung in der Deklaration von Helsinki haben, sowie in Übereinstimmung mit ICH-Good Clinical Practice, den geltenden gesetzlichen Bestimmungen und dem QM-Handbuch des Instituts für Sozialmedizin, Epidemiologie und Gesundheitsökonomie und deren SOPs und den SOPs der WALA Heilmittel GmbH durchgeführt.

## Genehmigung durch das BfArM

Die WALA Heilmittel GmbH reicht vor Beginn der klinischen Studie beim zuständigen BfArM einen Antrag auf Genehmigung der klinischen Studie ein. Die klinische Studie in einem Zentrum darf erst nach Vorliegen der Genehmigung des Antrags durch das BfArM begonnen werden.

Dem BfArM werden umgehend alle Änderungen zum Studienprotokoll sowie alle tödlich schwerwiegenden, schwerwiegenden unerwarteten sowie tödlich bzw. lebensbedrohlichen schwerwiegenden unerwarteten und im Zusammenhang mit dem Prüfpräparat stehenden unerwünschten Ereignisse, die während der klinischen Studie auftreten und die Sicherheit der Studienteilnehmer oder die Durchführung der Studie beeinträchtigen könnten, mitgeteilt.

Ein Annual Safety Report aller schwerwiegender Ereignisse mit kausalem Zusammenhang wird ein Jahr nach Erteilung der Genehmigung durch das BfArM an dieses gesendet.

## Bewertung durch die Ethikkommission

Vor Beginn der klinischen Prüfung legt die WALA Heilmittel GmbH die Unterlagen der klinischen Studie der zuständigen und beteiligten Ethikkommissionen der Studienärzte zur Bewertung vor. Die klinische Studie in einem Zentrum darf erst nach Vorliegen der zustimmenden Bewertung der zuständigen Ethikkommission begonnen werden.

Die WALA Heilmittel GmbH teilt der Ethikkommission umgehend alle Änderungen im Studienprotokoll mit, sowie tödlich bzw. lebensbedrohliche schwerwiegende unerwartete und im Zusammenhang mit dem Prüfpräparat stehende unerwünschten Ereignisse, die während der klinischen Studie auftreten und die die Sicherheit der Studienteilnehmer oder die Durchführung der Studie beeinträchtigen könnten.

Ein Annual Safety Report aller schwerwiegenden Ereignisse mit kausalem Zusammenhang wird ein Jahr nach Erteilung der Genehmigung durch das BfArM an die Ethikkommission gesandt.

Eventuelle Empfehlungen und Hinweise der Ethikkommission werden in das Studienprotokoll eingearbeitet.

Der Prüfarzt darf an der Entscheidung der Ethikkommission nicht mitwirken. Es wird eine Liste der Kommissionsmitglieder sowie die Satzung der Ethikkommission angefordert.

## Anzeige der klinischen Prüfung

Die WALA Heilmittel GmbH zeigt die klinische Prüfung nach §67 des Arzneimittelgesetzes bei den zuständigen Landesbehörden an.

## Patienteninformation und Einwilligungserklärung

Die Patienteninformation ist in einer für den Studienteilnehmer verständlichen Sprache und möglichst ohne Verwendung von Fachausdrücken geschrieben.

Der verantwortliche Prüfarzt eines jeden Prüfzentrums ist verpflichtet sicherzustellen, dass jeder Studienteilnehmer vor Studienbeginn mündlich und schriftlich über Wesen, Zweck, Behandlung, Bedeutung, mögliche Risiken und Nutzen sowie freiwillige Teilnahme an der klinischen Studie aufgeklärt wird. Der Studienteilnehmer muss die Möglichkeit haben, alle offenen Fragen zu klären und genügend Zeit und Gelegenheit haben, sich für oder gegen eine Teilnahme an der klinischen Studie zu entscheiden.

Der Studienteilnehmer sowie der Prüfarzt müssen die Einwilligungserklärung vor Beginn der studienspezifischen Untersuchungen eigenhändig datieren und unterschreiben. Der verantwortliche Prüfarzt eines jeden Prüfzentrums ist verpflichtet, das Original der Einwilligungserklärung an die Studienzentrale zu schicken, eine Kopie zu archivieren und eine Kopie dem Studienteilnehmer auszuhändigen.

Wenn Änderungen der Patienteninformation und Einwilligungserklärungen notwendig sind, wird eine neue Version beim BfArM und der zuständigen/ den beteiligten Ethikkommissionen eingereicht und die genehmigte Version an alle Studienzentren gesendet. Falls notwendig, werden die Studienteilnehmer erneut aufgeklärt und erneut um ihre schriftliche Einwilligung gebeten.

## Datenschutz

Personenbezogene Daten werden pseudonymisiert, d.h. unter Verwendung der Patientencodes dokumentiert und ausgewertet. Persönliche Daten werden an die Studienzentrale gesandt und dürfen darüber hinaus das Prüfzentrum nicht verlassen. Dokumente, durch die die Studienteilnehmer identifiziert werden können, wie z.B. die Patientenidentifikation oder die unterschriebene Einwilligungserklärung, werden beim Prüfarzt und der Studienzentrale aufbewahrt.

Vor der Aufnahme in die Studie müssen die Studienteilnehmer eine schriftlich Zustimmung zur Erfassung der persönlichen und studienbezogenen Daten, der Weitergabe in pseudonymisierter Form an die WALA Heilmittel GmbH, sowie zur Einsichtnahme der Gesundheitsdaten und der personenbezogenen Daten durch autorisierte und zur Verschwiegenheit verpflichtete Beauftragte des Sponsors sowie die zuständigen inländischen Überwachungsbehörden unterschreiben.

Alle Personen, die in die Dokumentation der erhobenen Befunde und in die Einsichtnahme und Überprüfung der persönlichen Daten eingebunden sind, sind zur strikten Vertraulichkeit verpflichtet.

Die Beachtung des Bundesdatenschutzgesetzes und des Landesdatenschutzgesetzes ist in vollem Umfang gewährleistet.

Vor Beginn der Studie wird die Genehmigung des Datenschutzbeauftragten des Instituts für Sozialmedizin, Epidemiologie und Gesundheitsökonomie und des Datenschutzbeauftragten der Charité eingeholt.

## Versicherung

In Übereinstimmung mit GCP schließt das Institut für Sozialmedizin, Epidemiologie und Gesundheitsökonomie der Charité - Universitätsmedizin Berlin im Auftrag der WALA Heilmittel GmbH zugunsten aller Studienteilnehmer eine Versicherung ab mit dem

*Gerling Konzern*

*Allgemeine Versicherungs-AG*

*GIS / Liability*

*Gereonshof 8*

*50597 Köln*

*Probanden-Jahresvertrag Nr. 70-5644584-4*

*Anmeldenummer: 1600200630*

Die Studienteilnehmer werden vom Prüfarzt über die Versicherung und die damit verbundenen Rechte und Pflichten informiert und erhalten eine Kopie der Versicherungsbescheinigung und -bedingungen. Gemäß den Versicherungsbedingungen ist der Studienteilnehmer verpflichtet, jeglichen gesundheitlichen Schaden, der Folge der klinischen Studie sein könnte, unverzüglich an den Prüfarzt und die Versicherung zu melden.

## Finanzielle Angelegenheiten

Die vorliegende Studie wird von der WALA Heilmittel GmbH finanziert. Kostenerstattungen/ Aufwandsentschädigungen und Honorare für Studienärzte werden in gesonderten Vereinbarungen geregelt. Die Studienteilnehmer erhalten kein Honorar.

## Studienabschlussbericht / Publikation

Nach Beendigung (auch bei vorzeitigem Ende) der Studie wird in Anlehnung an die ICH-Guideline “Structure and Content of Clinical Study Reports“ und unter Berücksichtigung der Studienziele ein finalisierter Abschlussbericht im Einvernehmen mit der WALA Heilmittel GmbH von der Studienzentrale erstellt. Eine Publikation ist vorgesehen. Studienabschlussbericht und Publikationsrechte werden in einer gesonderten Vereinbarung geregelt.

# Vorgehensweise bei Notfall, Überdosierung oder Schwangerschaft

## Notfall

In einem Notfall liegt es in der Verantwortung des verantwortlichen Prüfarztes eines jeden Prüfzentrums, die medizinische Versorgung der Studienteilnehmer zu sichern. Alle SUEs sind zu dokumentieren und innerhalb von 24h an die WALA Heilmittel GmbH zu melden.

## Überdosierung, Schwangerschaft

Im Fall einer Überdosierung ist die medizinische Versorgung des Prüfungsteilnehmers zu sichern. Die Überdosierung ist zu dokumentieren.

Im Falle einer Schwangerschaft ist diese zu dokumentieren und zu verfolgen. Die Therapie wird für diese Patientin abgebrochen.

# Literatur

1. Kohlmann T, Raspe H. Hannover Functional Questionnaire in ambulatory diagnosis of functional disability caused by backache. *Rehabilitation* 1996; **35:** I-VIII.

2. Bullinger M, Kirchberger I. SF-36 Fragebogen zum Gesundheitszustand. Göttingen: Hogrefe, 1998.

3. Geissner E, Schulte A. Die Schmerzempfindungsskala (SES). Göttingen: Hogrefe, 1996.

4. Dillmann U, Nilges P, Saile H, Gerbershagen HU. Behinderungseinschätzung bei chronischen Schmerzpatienten. *Schmerz* 1994; **8:** 100-10.

5. Huskisson EC. Measurement of pain. *Lancet* 1974; **2:** 1127-31.

6. Koes BW, van Tulder MW, Thomas S. Diagnosis and treatment of low back pain. *BMJ* 2006; **332:** 1430-4.

7. Speed C. Low back pain. *BMJ* 2004; **328:** 1119-21.

8. Andersson G. The epidemiology of spinal disorders. In: Frymoyer JW., editor. The adult spine: principles and practice.Philadelphia: Lippincott-Raven, 1997. p. 93-141.

9. Picavet HSJ., Schouten JSAG., Smith HA. Prevalences and consequences of low back pain in the MORGEN-projekt 1993-1995. Bilthoven, Netherlands: Rijksinstituut voor Volksgezondheid en Milieu, 1996.

10. Thomas KJ, Fitter M, Brazier J, MacPherson H, Campbell M, Nicholl JP, et al. Longer-term clinical and economic benefits of offering acupuncture to patients with chronic low back pain assessed as suitable for primary care management. *Complement Ther Med* 1999; **7:** 91-100.

11. Cherkin DC, Deyo RA, Wheeler K, Ciol MA. Physician variation in diagnostic testing for low back pain. Who you see is what you get. *Arthritis Rheum* 1994; **37:** 15-22.

12. Cherkin DC, Deyo RA, Loeser JD, Bush T, Waddell G. An international comparison of back surgery rates. *Spine* 1994; **19:** 1201-6.

13. van Tulder MW, Koes BW. Low back pain: chronic. Clinical Evidence. London: BMJ Publishing Group, 2006.

14. Deyo RA, Weinstein JN. Low back pain. *N Engl J Med* 2001; **344:** 363-70.

15. Eisenberg DM, Davis RB, Ettner SL, Appel S, Wilkey S, Van Rompay M, et al. Trends in alternative medicine use in the United States, 1990-1997: results of a follow-up national survey. *JAMA* 1998; **280:** 1569-75.

16. Härtel U, Volger E. Inanspruchnahme und Akzeptanz klassischer Naturheilverfahren und alternativer Heilmethoden in Deutschland - Ergebnisse einer repräsentativen Bevölkerungsstudie. *Forsch Komplementärmed Klass Naturheilkd* 2004; **11:** 327-34.

17. Thomas KJ, Nicholl JP, Coleman P. Use and expenditure on complementary medicine in England: a population based survey. *Complement Ther Med* 2001; **9:** 2-11.

18. Manheimer E, White A, Berman B, Forys K, Ernst E. Meta-analysis: acupuncture for low back pain. *Ann Intern Med* 2005; **142:** 651-63.

19. van Tulder MW, Furlan AD, Gagnier JJ. Complementary and alternative therapies for low back pain. *Best Pract Res Clin Rheumatol* 2005; **19:** 639-54.

20. Gagnier JJ, van TM, Berman B, Bombardier C. Herbal medicine for low back pain. *Cochrane Database Syst Rev* 2006; **19;** CD004504.

21. Gerbershagen U. Organized treatment of pain. Determination of status. *Internist* 1986; **27:** 459-69.

22. Vincent C. Credibility assessments in trials of acupuncture. *Complementary Medical Research* 1990; **4:** 8-11.

23. Schmerzfragebogen. www.medizin.uni-koeln.de/projekte/dgss/Schmerzfragebogen html 2002 March 28.

# Anhänge

Anhang 1 Muster der Patienteninformation

Anhang 2 Muster der Einwilligungserklärung

Anhang 3 Unterschriften LKP und Sponsor

Anhang 4 Erklärung des Prüfarztes
